# Mitochondrial and Plastid Genomes of the Colonial Green Alga *Gonium pectorale* Give Insights into the Origins of Organelle DNA Architecture within the Volvocales

**DOI:** 10.1371/journal.pone.0057177

**Published:** 2013-02-26

**Authors:** Takashi Hamaji, David R. Smith, Hideki Noguchi, Atsushi Toyoda, Masahiro Suzuki, Hiroko Kawai-Toyooka, Asao Fujiyama, Ichiro Nishii, Tara Marriage, Bradley J. S. C. Olson, Hisayoshi Nozaki

**Affiliations:** 1 Department of Botany, Graduate School of Science, Kyoto University, Oiwake-cho, Kita-shirakawa, Sakyo-ku, Kyoto, Japan; 2 Canadian Institute for Advanced Research, Department of Botany, University of British Columbia, Vancouver, British Columbia, Canada; 3 Center for Advanced Genomics, National Institute of Genetics, Mishima, Shizuoka, Japan; 4 Center for Information Biology, National Institute of Genetics, Mishima, Shizuoka, Japan; 5 Department of Biological Sciences, Graduate School of Science, University of Tokyo, Hongo, Bunkyo-ku, Tokyo, Japan; 6 Temasek Life Sciences Laboratory, The National University of Singapore, Singapore, Singapore; 7 Division of Biology, Kansas State University, Manhattan, Kansas, United States of America; University of Melbourne, Australia

## Abstract

Volvocalean green algae have among the most diverse mitochondrial and plastid DNAs (mtDNAs and ptDNAs) from the eukaryotic domain. However, nearly all of the organelle genome data from this group are restricted to unicellular species, like *Chlamydomonas reinhardtii*, and presently only one multicellular species, the ∼4,000-celled *Volvox carteri*, has had its organelle DNAs sequenced. The *V. carteri* organelle genomes are repeat rich, and the ptDNA is the largest plastome ever sequenced. Here, we present the complete mtDNA and ptDNA of the colonial volvocalean *Gonium pectorale*, which is comprised of ∼16 cells and occupies a phylogenetic position closer to that of *V. carteri* than *C. reinhardtii* within the volvocine line. The mtDNA and ptDNA of *G. pectorale* are circular-mapping AT-rich molecules with respective lengths and coding densities of 16 and 222.6 kilobases and 73 and 44%. They share some features with the organelle DNAs of *V. carteri*, including palindromic repeats within the plastid compartment, but show more similarities with those of *C. reinhardtii*, such as a compact mtDNA architecture and relatively low organelle DNA intron contents. Overall, the *G. pectorale* organelle genomes raise several interesting questions about the origin of linear mitochondrial chromosomes within the Volvocales and the relationship between multicellularity and organelle genome expansion.

## Introduction

Some of the most diverse and bizarre organelle genomes of all eukaryotes come from the Volvocales, which is a large order of predominantly freshwater green algae, belonging to chlorophycean class of the Chlorophyta. Volvocalean mitochondrial and plastid DNAs (mtDNAs and ptDNAs) show an impressive array of architectures, nucleotide landscapes, and coding compositions (Table 1)– and see Leliaert et al. [1] and Lee and Hua [2] for additional compilations. Moreover, certain volvocalean species, particularly those within the “*Reinhardtinia* clade” *sensu* Nakada et al. [3], have proven to be excellent systems for testing contemporary hypotheses on the evolution of organelle genome expansion and linearization [4], [5], [6].

**Table 1:** Completely sequenced organelle genomes from volvocalean green algae.

Species	Clade (lineage)	Organelle genome architecture						
		MappingConformation	Size(kb)	AT content(%)	Coding(%)	Protein-codinggenes	Introns	GenBank/DDBJAccession
MITOCHONDRIAL DNA								
* Chlamydomonas* *reinhardtii*	*Reinhardtinia*(volvocine)	Linear	16–19	55	67–82	7	0–3	EU306617–23
* Chlamydomonas* *moewusii*	*Xenovolvoxa*	Circular	23	65	54	7	9	AF008237
* Chlorogonium* *elongatum*	*Caudivolvoxa*	Circular	23	62	53	7	6	Y13643–4,Y07814
* Dunaliella salina*	*Caudivolvoxa*	Circular	28	66	42	7	18	GQ250045
* Gonium pectorale*	*Reinhardtinia*(volvocine)	Circular	16	61	73	7	1	AP012493
* Polytomella capuana*	*Reinhardtinia*	Linear	13	43	82	7	0	EF645804
* Polytomella parva*	*Reinhardtinia*	Linear	16	59	66	7	0	AY062933-4
* Polytomella* sp.SAG 63–10	*Reinhardtinia*	Linear	16	58	66	7	0	GU108480-1
* Volvox carteri*	*Reinhardtinia*(volvocine)	Circular	35	66	<40	7	3	EU760701,GU084821
PLASTID DNA								
* Chlamydomonas* *reinhardtii*	*Reinhardtinia*(volvocine)	Circular	204	66	44	66	7	FJ423446
* Dunaliella salina*	*Caudivolvoxa*	Circular	269	68	35	66	>35	GQ250046
* Gonium pectorale*	*Reinhardtinia*(volvocine)	Circular	223	70	44	66	3	AP012494
* Volvox carteri*	*Reinhardtinia*(volvocine)	Circular	525	57	<20	66	9	GU084820

Note: Values rounded to whole numbers. Clade names are based on Nakada et al. [3]. Percent coding includes all annotated protein-, rRNA-, and tRNA-coding regions as well as non-standard ORFs, such as the *rtl* gene in the *C. reinhardtii* mtDNA. Gene number includes standard protein-coding genes, but does not include intronic or nonstandard ORFs, like *rtl*. Duplicate genes and introns were counted only once. Genome statistics for *P. parva* and *P. piriformis* are based on the concatenation of the two mitochondrial chromosomes; those for *V. carteri* should be considered as approximations as the mtDNA and ptDNA contain assembly gaps due to unresolved repeats. For *C. reinhardtii*, the mitochondrial genome size, intron number, and coding content can vary because of optional introns.

Most of our understanding of volvocalean mitochondrial and plastid genomes is limited to unicellular species, such as the model organisms *Chlamydomonas reinhardtii* and *C. globosa* (previously misidentified as *C. incerta*; see Nakada et al. [7]) [5], [8], the colorless and wall-less *Polytomella capuana*, *P. parva*, and *P. piriformis* [6], [9], [10], and the halotolerant β-carotene-rich *Dunaliella salina* [11]. Surprisingly little is known about the organelle genomes of colonial and multicellular volvocaleans, which are found within the volvocine lineage of the *Reinhardtinia* clade (Figure S1). Volvocine algae are preeminent models for studying the evolution of multicellularity [12], [13], and span the gamut of cellular complexity, from simple 4-celled species (e.g., *Tetrabaena*), to 8-64-celled colonial forms (e.g., *Gonium*), all the way to highly complex spheroidal taxa, with more than 500 cells (e.g., *Volvox*) [14], [15]. It is estimated that multicellular volvocine species last shared a common unicellular ancestor ∼200 million years ago [15].

Of the 8 different volvocalean algae for which complete mtDNA and/or ptDNA sequences are available [11], only one is multicellular: *Volvox carteri*, which is comprised of ∼4,000 cells. The organelle genomes of this species are distended with repetitive noncoding DNA, and similar palindromic repeats are located in both the mitochondrial and plastid compartments [16]. Moreover, the *V. carteri* ptDNA, at ∼525 kb, is among the largest plastomes ever observed (from any eukaryote) [4], dwarfing that of *C. reinhardtii*, which is 204 kb [17]. Although smaller than its plastid counterpart, the ∼35 kb mtDNA of *V. carteri* is still larger than any the other completely sequenced volvocalean mitochondrial genome. It is hypothesized that the expanded organelle genomes of *V. carteri* are a consequence of a low organelle mutation rate and/or a small effective population size [4].

The *V. carteri* mtDNA assembles as a circular molecule, contrasting the linear (or linear fragmented) architectures of all other well-studied *Reinhardtinia*-clade mitochondrial genomes, including those of *C. reinhardtii*, *Polytomella* spp., and the multicellular *Pandorina morum* [5], [11], [18]. These linear mtDNAs have evolved complex terminal structures [5], [10], called mitochondrial telomeres, which form long palindromic repeats at the genome ends. The origin and number of times that linear mitochondrial chromosomes have evolved within the *Reinhardtinia* is unknown, but it has been argued that they arose only once [4]. If true, this would imply that in a recent ancestor of *V. carteri*, the mtDNA reverted from a linear to a circular form.

To learn about organelle genome architecture within multicellular volvocine algae and to gain insight into ptDNA expansion and the origin of linear mtDNAs, we sequenced the mitochondrial and plastid genomes of *Gonium pectorale*–an 8- or 16-celled freshwater colonial alga, occupying a phylogenetic position closer to that of *V. carteri* than *C. reinhardtii* within the volvocine line [14], [15], [19] (Figure S1).

## Materials and Methods

The organelle genomes described here come from *Gonium pectorale* K3-F3-4 (mating type *minus*), which was one of the F3 backcross strains to K41 (mating type *plus*) (originating from K41×K32 [F1 strains of Kaneko3×Kaneko4]) [20], [21] and is available as NIES-2863 from the Microbial Culture Collection at National Institute for Environmental Studies, Tsukuba, Japan (http://mcc.nies.go.jp/). *G. pectorale* was grown in 200–300 mL VTAC medium [22], [23] at 20°C on a 14∶10 h light-dark cycle, under cool-white fluorescent lamps (165–175 µmol m^−2^ s^−1^ intensity). Total DNA was extracted based on the protocol of Miller et al. [24].

Sequencing libraries were prepared from *G. pectorale* K3-F3-4 genomic DNA using the GS FLX Titanium Rapid Library Preparation Kit (F. Hoffmann-La Roche, Basel, Switzerland) and the TruSeq DNA Sample Prep Kit (Illumina Inc., San Diego, CA, USA), and were run on a GS FLX (F. Hoffmann-La Roche) and a MiSeq sequencer (Illumina Inc.), respectively. The GS FLX reads were assembled with Newbler v2.6. A fosmid library (23,424 clones) was constructed from *G. pectorale* K3-F3-4 genomic DNA using fosmid vector pKS300, which was developed in-house. End sequencing of the fosmid library and the BAC library of *G. pectorale* Kaneko3 (18,048 clones, Genome Institute (CUGI), Clemson Univ., Clemson, SC, USA) was carried out using a BigDye terminator kit ver3 (Life Technologies, Carlsbad, California, USA) and was run on automated ABI 3730 capillary sequencers (Life Technologies). The GS FLX contig sequences, which were derived from mitochondrial and chloroplast genomes, and the BAC/fosmid end-sequences were assembled using the Phrap/Consed systems. Gap closing and re-sequencing of low-quality regions in the assembly were performed by shotgun sequencing of the corresponding BAC/fosmid clones, PCR, primer walking, and direct sequencing of fosmid clones. The MiSeq sequence reads were mapped against the assembly sequences using the BWA program [25] after passing through the quality filter. The errors on each GS FLX assembly sequence were also corrected. The assembling delineated one circular mtDNA and two ptDNA isoforms (A and B), a common feature of plastid genomes with inverted repeats [26], [27] (Figure S2). The annotated *G. pectorale* mtDNA and ptDNA (isoform A) sequences are deposited in the DDBJ database under accession numbers AP012493 and AP012494, respectively.

Phylogenetic analyses were performed under maximum likelihood (ML) using RAxML [28] and PhyML 3.0 [29] with 100 bootstrap replicates. Maximum parsimony (MP) bootstrap analyses (based on 10 random replications of the full heuristic search with the tree bisection–reconnection branch-swapping algorithm) were performed in PAUP 4.0b10 [30] with 1,000 replications. MtDNA protein phylogeny was based on the deduced *nad5*, *cox1*, and *cob* amino acid sequences (Table S1), which were aligned using Clustal X [31]. Intron phylogenies were based on the deduced and aligned amino acid sequences of the *nad5* and *psaB* intronic open reading frames (ORFs), which gave data matrices of 205 and 256 amino acids with 9 and 14 operational taxonomic units (OTUs), respectively (Tables S2, S3). Intron secondary structure maps were constructed as previously described [32].

## Results and Discussion

### The *Gonium pectorale* mtDNA: A Compact Circular Mapping Chromosome

**Figure 1:**
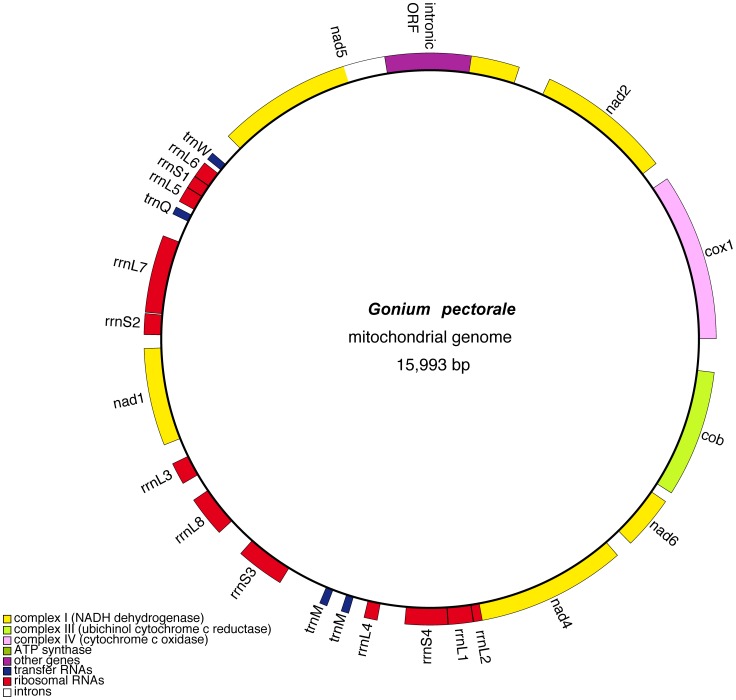
Genetic map of the *Gonium pectorale* mitochondrial genome. Note, the *G. pectorale* mtDNA is a circular-mapping molecule. Transfer RNA-coding regions are designated by the single-letter abbreviation of the amino acid they specify.

The mitochondrial genome of *G. pectorale* has a conservative architecture: it is small (16 kb), circular-mapping, AT rich (61%), compact (73% coding), contains very few repeats, and has only a single intron (Figure 1, Table 1, Figure S3). It lacks the eccentricities that often characterize the mtDNAs of other volvocalean species, such as a high GC content (e.g., *P. capuana*), a linear or linear-fragmented conformation (e.g., *P. parva*), a large intron density (e.g., *D. salina*), non-standard genes (e.g., *C. reinhardtii*), and/or a bloated repeat-rich structure (e.g., *V. carteri*) [11]. The *G. pectorale* mtDNA is gene poor, encoding 7 proteins, 2 rRNAs, and 3 unique tRNAs, representing methionine, glutamine, and tryptophan (Figure 1). Two copies of *trnM* were identified adjacent to one another in the genome. Both have similar sequences and cloverleaf structures, and appear to have a role in elongation rather than initiation, as suggested for the *trnM* of other volvocalean algae. When ignoring non-standard genes and duplicate tRNAs, the *G. pectorale* mitochondrial gene repertoire mirrors those from all other available volvocalean algae, with the exception of *Polytomella* species, which lack *trnW* and *trnQ*. The *G. pectorale* mitochondrial large and small subunit (LSU and SSU) rRNA genes, like those from other available *Reinhardtinia* algae, are fragmented and scrambled throughout the genome into 8 and 4 coding modules, respectively. In *V. carteri* the eighth LSU module has been invaded by palindromic repeats, splitting it into two segments (L8a and L8b) [16]; in *G. pectorale*, however, the L8 module is intact.

The sole intron of the *G. pectorale* mtDNA, located in *nad5*, is of group ID affiliation [33] (Figure S3) and encodes a putative intronic endonuclease. Other volvocaleans contain a *nad5* group I intron (with the same insertion site), but none are from the *Reinhardtinia* clade. Our phylogenetic analyses of various volvocalean intronic ORFs (Figure S4) suggest that the *G. pectorale nad5* intron either was acquired through horizontal transmission from a volvocalean closely related to *Chlamydomonas moewusii* or *Chlorogonium elongatum* or that it was present in the ancestor of the Volvocales and preserved in *G. pectorale*.

Linear mitochondrial chromosomes are widespread throughout the *Reinhardtinia* clade, occurring in all explored taxa [34], with the exception of *V. carteri*, which has a circular mtDNA map, but rare possible linear forms of the genome have been observed [4], [16] (Table 1). Our *de novo* and mapping assemblies of the *G. pectorale* mtDNA gave an unambiguous circular-mapping chromosome (see Materials and Methods), and although such a map could represent a circularly permuted, linear-type structure, various features of the *G. pectorale* mitochondrial genome support the idea that it is circular. For instance, all twelve of the *G. pectorale* mtDNA genes have the same transcriptional polarity–a trait that is also found in *V. carteri* and available volvocalean species with circular mitochondrial genomes. Conversely, in all of the sequenced linear mtDNAs from the Volvocales, the genes are divided into two transcriptional polarities, proceeding outward towards the ends of the chromosome [6].Furthermore, our Southern blot analysis of the *G. pectorale* mtDNA, cut with restriction enzymes, demonstrates that it is a circular molecule (Figure S5).

**Figure 2:**
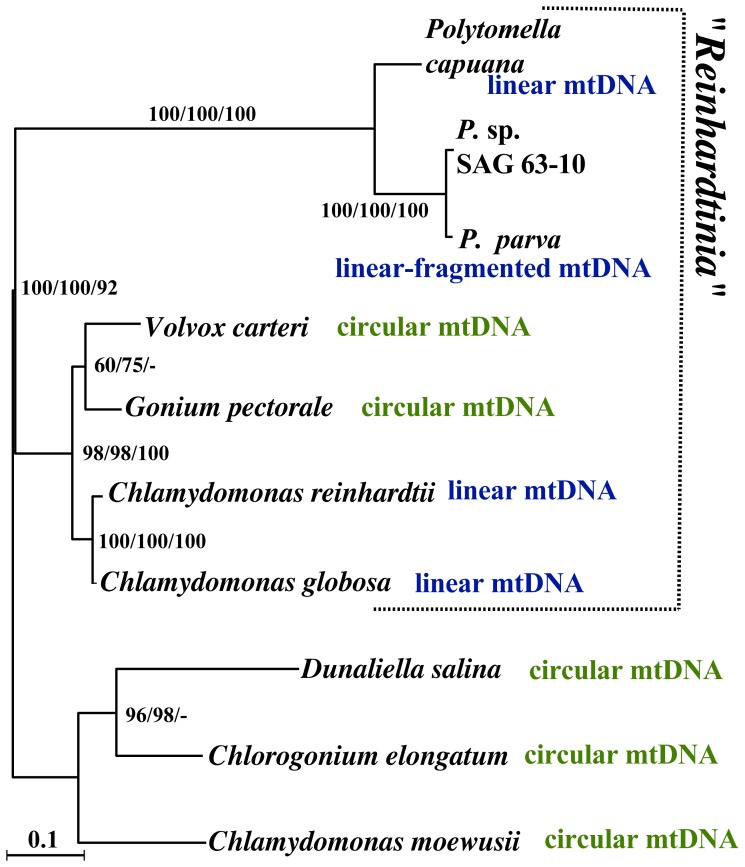
MtDNA protein phylogeny of seven species belonging to *Reinhardtinia* clade and three outgroup species from the Volvocales. The tree was constructed under the RAxML (with WAG+I+4G model) method using the concatenated sequences of the deduced *nad5*, *cox1*, and *cob* amino acid sequences. Left, middle, and right bootstrap values (≥50%) obtained using the RAxML, PhyML (with LG+I+4G model), and MP analysis, respectively. The amino acid sequences of the three proteins were aligned by Clustal X [29], and ambiguously aligned and highly variable regions were removed to construct a multiprotein data matrix of 909 amino acids from the 10 operational taxonomic units (Table S1).

Our evidence for a circular mitochondrial genome in *G. pectorale* raises interesting questions about the origin of linear mtDNAs within the *Reinhardtinia* clade. There is little doubt that the ancestral volvocalean mtDNA was circular, and it is argued that there was a single shift from a circular to a linear mtDNA structure in the ancestor that gave rise to *Reinhardtinia* algae [6]. Within the *Reinhardtinia* clade, *V. carteri* and *G. pectorale* belong to a monophyletic colonial or multicellular volvocalean group from which unicellular members are separated [14], [15] (Figure 2), but the multicellular volvocalean *Pandorina morum* has a linear mtDNA [18]. Moreover, *V. carteri* and *P. morum* belong to the monophyletic Volvocaceae from which *G. pectorale* is excluded [14], [15], [19] (Figure S1). Thus, the appearance of circular mitochondrial genome maps in both *V. carteri* and *G. pectorale* suggests that the mtDNAs of these species independently reverted from a linear to a circular conformation in the two separate ancestors of *G. pectorale* and *V. carteri* (Figure S1) or alternatively that there were multiple origins of linear mitochondrial genomes in the *Reinhardtinia* clade, in the ancestors of *Polytomella*, *C. reinhardtii*, and *P. morum* (Figure S1). Studies of mtDNA structure from other volvocine species, such as *Tetrabaena* and *Yamagishiella*, are needed to further investigate these hypotheses.

### The *Gonium pectorale* ptDNA Shows Moderate Genome Expansion

Volvocalean plastid genomes are big and that of *G. pectorale*, at 222.6 kb, is no exception. Of the approximately 300 complete (or almost complete) ptDNAs in GenBank, as of 1 August 2012, fewer than ten have a length >200 kb, all but one of which are from chlorophyte green algae, including the volvocaleans *C. reinhardtii* (204 kb), *D. salina* (269 kb) and *V. carteri* (∼525 kb) [4], [11], [17]. The large size of volvocalean ptDNAs is not a product of an inflated gene number, but a consequence of having an abundance of noncoding nucleotides, often represented by repetitive elements and introns. This is also true for the *G. pectorale* ptDNA, which is 56% (∼125 kb) noncoding. Almost all of these noncoding nucleotides are AT rich (average = 71%) and found in intergenic regions.

**Figure 3:**
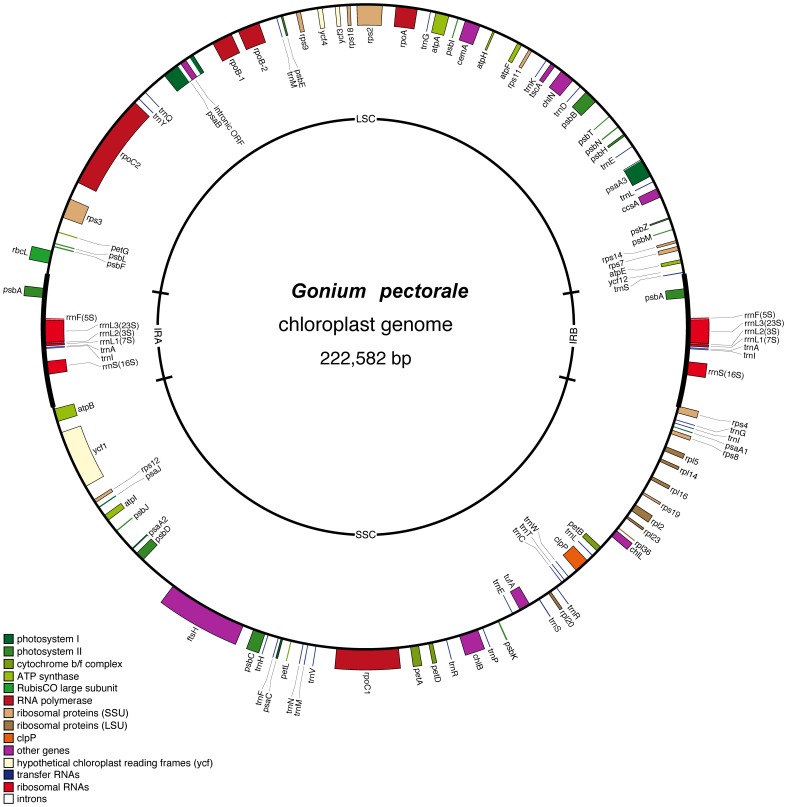
Genetic map of the *Gonium pectorale* plastid genome. Note, the *G. pectorale* ptDNA is a circular-mapping molecule. Transfer RNA-coding regions are designated by the single-letter abbreviation of the amino acid they specify.

The coding regions also have a high AT content (68%) and encompass a total of 98 unique genes, encoding 67 proteins, 3 rRNAs, 27 tRNAs, and a single misc RNA (*tscA*) (Figure 3). Six of these genes (*psbA*, *rrnL*, *rrnS*, *rrnF*, *trnA,* and *trnI*) are duplicated, being located in a pair of 14.8 kb inverted repeats, which divide the *G. pectorale* ptDNA into a large (99.6 kb) and a small (93.5 kb) single-copy region (Figure 3). This gene complement and inverted-repeat arrangement is almost identical to those of *C. reinhardtii* and *V. carteri* (Figure 3, Figure S6).

Although some volvocalean algae harbour many ptDNA introns (Table 1)–*V. carteri* has 9 and *D. salina* has >35–*G. pectorale* harbours just three: one located in *psaB*, which appears to be of group IA affiliation [33] (Figure S3), and encodes a putative endonuclease-like protein, and two short group II introns (117 and 176 bp) found upstream of *psaA* exons 2 and 3 (Figure 3). Phylogenetic analysis of the *G. pectorale* intron (Figure S7) show that it is closely related to the *psaB* group I intron of the chlorophycean (but non-volvocalean) green alga *Stigeoclonium helveticum* [35]; moreover, both introns have the same insertion site within the *psaB* gene. *V. carteri* also has a *psaB* intron, but it is of group II affiliation [4]. In fact, there is not a single homologous pair of either group I or group II introns among the *G. pectorale*, *V. carteri*, and *C. reinhardtii* plastid genomes (Figure S6), suggesting that rapid horizontal intron transfer and loss occurred within the colonial Volvocales.

The *G. pectorale* plastid genome, like its *V. carteri* and *C. reinhardtii* counterparts, contains hundreds of short repetitive elements, distributed throughout the intergenic regions, as demonstrated by the dotplot similarity matrix (Figure S8). Many of the *V. carteri* ptDNA repeats are palindromes, and can be folded into hairpin structures [16]. The same is true for the *G. pectorale* ptDNA, which contains ∼135 short (13 nt) palindromic repeats (including eight in the coding regions) with the motif: 5′- TCCCCNNNGGGGA-3′ (Figure S9). This is fewer repeats than found in the *V. carteri* ptDNA, which contains over a thousand palindromic elements.

The *G. pectorale* ptDNA is slightly more expanded (by ∼19 kb) than that of *C. reinhardtii*, but much smaller than those of the unicellular *D. salina* (269 kb, ∼65% noncoding) and the multicellular *V. carteri* (∼525 kb, >80% noncoding) (Table 1). What has led to such a wide spectrum of ptDNA expansion within the Volvocales? One contemporary–and controversial [36], [37]–hypothesis for the evolution of genome size, called the mutational hazard hypothesis [38], argues that genome expansion is a product of a low effective population size (*N_e_*) (which results in increased random genetic drift) and/or a low mutation rate (μ), which reduces the burden of harbouring excess DNA. The *V. carteri* ptDNA is estimated to have a very low *N_e_*μ [4], about twenty times lower than that of the *C. reinhardtii* ptDNA [39], which may explain why it is so bloated. We do not know the value of *N_e_*μ for the *G. pectorale* ptDNA–this will require sequencing the plastid genomes of several additional *G. pectorale* isolates. However, given that this species is ∼10 times larger than *C. reinhardtii* (16 cells vs a single cell) and a hundred times smaller than *V. carteri* (16 cells vs 4,000 cells), and that all three of these algae are found in a similar environment (freshwater ponds)–unlike *D. salina*, which is marine–one might expect the effective population size of *G. pectorale* to be similar or marginally smaller than that of *C. reinhardtii*, and much larger than that of *V. carteri*. If true, this may have contributed to *G. pectorale* having a ptDNA architecture comparable to that of *C. reinhardtii* but much different than that of *V. carteri*. Under this hypothesis, it can therefore be predicted that as more volvocine organelle DNAs are sequenced, species with large cell numbers and presumably low effective population sizes will have more bloated genomes than those with small cell numbers and large effective population sizes.

## Supporting Information

Figure S1 Simplified diagram for phylogenetic relationships of selected taxa of the unicellular, colonial and multicellular vovlocaleans.
(TIF)

Figure S2 Diagrams of possible isoforms of ptDNA of *Gonium pectorale*. A. Two isoforms as found in other ptDNAs with a typical inverted repeat. B. Two additional isoforms that were not rejected based on assembling of our sequence data.
(TIF)

Figure S3 Secondary structures of group I introns within the *Gonium pectorale* organelle DNAs. A. Mitochondrial *nad5* group ID intron. B. Chloroplast *psaB* group IA intron.
(TIF)

Figure S4 Phylogeny of *Gonium pactorale nad5* group I intronic ORF. The tree was constructed under the RAxML (with WAG+4G model) method using 8 additional, related amino acid sequences selected based on the topology of the distance tree provided by blastp research of NCBI (http://www.ncbi.nlm.nih.gov/). Numbers on the left, middle and right at branches represent bootstrap values (≥50%) obtained using the RAxML, PhyML (with LG+4G model), and MP analysis, respectively. The amino acid sequences were aligned by Clustal X, and ambiguously aligned and highly variable regions were removed to construct a data matrix of 205 amino acids from the 9 operational taxonomic units (Table S2).
(TIF)

Figure S5 Southern blot analysis of *Gonium pectorale* mtDNA with four restriction enzymes that cut the genome once (SacI and StuI) or twice (SacII and EcoRI). Genome map coordinates are based on the *G. pectorale* mtDNA DDBJ accession (AP012493). SacI and StuI digestions each gave single genome-sized bands (∼16 kb), and the SacII and EcoRI reactions each gave two bands. These data are consistent with the *G. pectorale* mtDNA being a circular molecules. Probe DNA was amplified by PCR with two specific primers (Gopec-mito-F 5′-CGGGCAAAGCATAATTAGTGTAG-3′ and Gopec-mito-R 5′-ACGAACAAGAGGAAGACCTAAC-3′).
(TIF)

Figure S6 Venn diagram comparing the gene repertoires of three volvocalean chloroplast genomes (AP012494, GU084820 and FJ423446). 102 genes (single asterisk) shared by the three genomes include 12 genes distributed in IRA and IRB and *trnI* (cau), which was previously annotated as one of the triplicated *trnM* in *C. reinhardtii* and *V. carteri*. Double asterisks represent one of the duplicated genes in *G. pectorale* and *C. reinhardtii*. Triple asterisks exhibit one of the duplicated genes in *C. reinhardtii*. Note that all intronic ORFs in *G. pectprale* (1^#^) and *V. carteri* (6^#^) are unique for each genome and considered “non-coding” in the text.
(PDF)

Figure S7 Phylogeny of *Gonium pactorale psaB* group I intronic ORF. The tree was constructed under the RAxML (with WAG+4G model) method using 13 additional, related amino acid sequences selected based on the topology of the distance tree provided by blastp research of NCBI (http://www.ncbi.nlm.nih.gov/). Numbers on the left, middle and right at branches represent bootstrap values (≥50%) obtained using the RAxML, PhyML (with LG+G model), and MP analysis, respectively. The amino acid sequences were aligned by Clustal X, and ambiguously aligned and highly variable regions were removed to construct a data matrix of 256 amino acids from the 14 operational taxonomic units (Table S3).
(TIF)

Figure S8 Dotplot similarity matrix of the *Gonium pectorale* plastid genome. The X- and Y-axes each represent the *G. pectorale* plastid genome (222.6 kb). Dots in the nucleotide similarity matrix represent regions of sequence similarity. The matrix was generated using JDotter, with a sliding-window size of 50. The inverted repeats are highlighted in red in the matrix.
(TIF)

Figure S9 Distribution of short (13 nt) palindromic repeats (including seven [red arrows] in five coding regions [blues arrows]) with the motif: 5′- TCCCCNNNGGGGA-3′ in ptDNA of *Gonium pectorale*. The repeats were examined by using Serial Cloner 2.5 (http://serialbasics.free.fr/Serial_Cloner.html).
(JPG)

Table S1 Amino acid alignment and origin of the data used for Figure 2.
(DOC)

Table S2 Amino acid alignment and origin of the data used for Figure S4.
(DOC)

Table S3 Amino acid alignment and origin of the data used for Figure S7.
(DOC)
