# Supplementary figures and images for: Mitochondrial and Plastid Genomes of the Colonial Green Alga Gonium pectorale Give Insights into the Origins of Organelle DNA Architecture within the Volvocales
Source: PLoS One. 2013 Feb 26;8(2):e57177. doi: 10.1371/journal.pone.0057177 (PMC3582580; doi:10.1371/journal.pone.0057177)

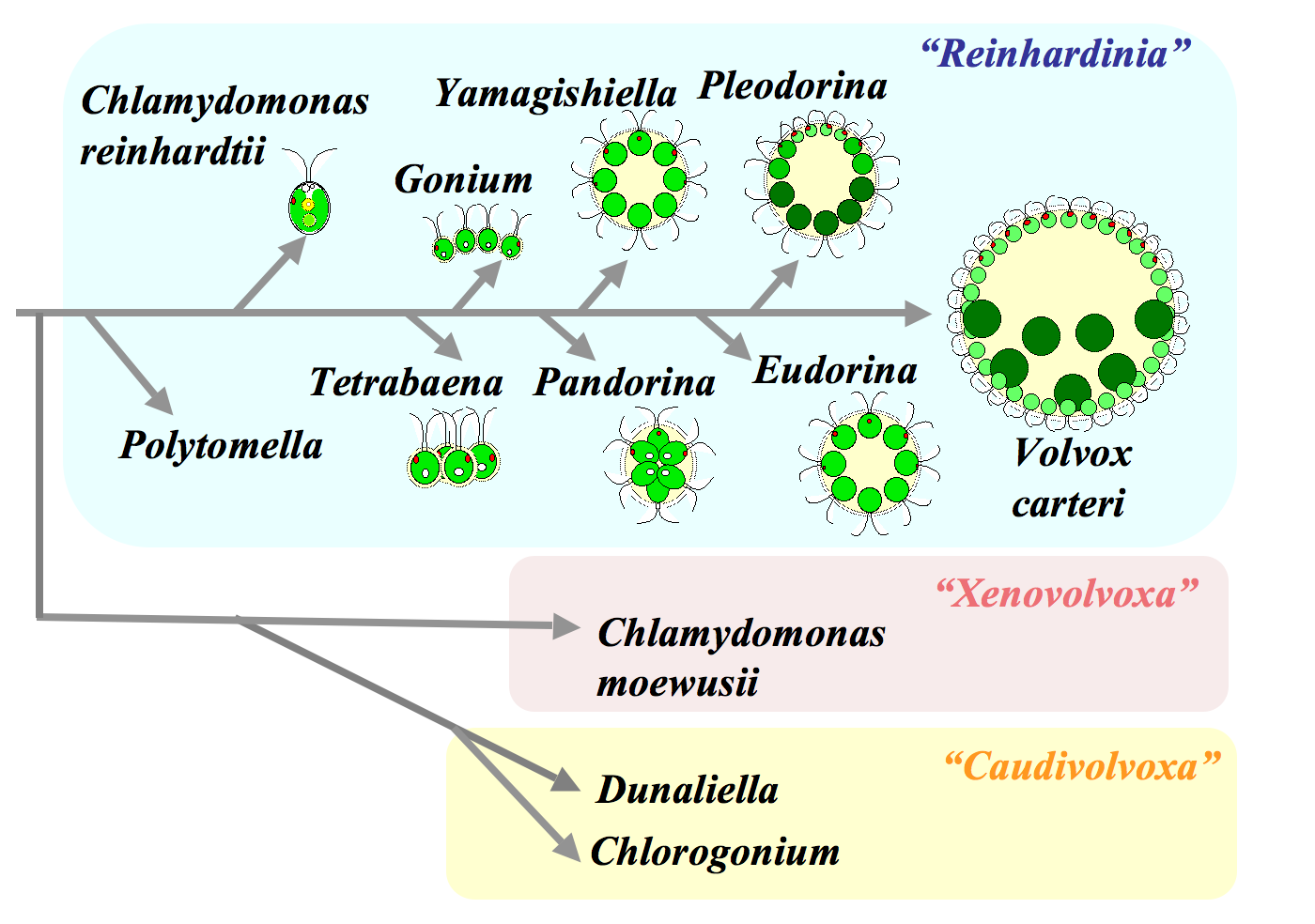

Supplement: Figure S1 — Simplified diagram for phylogenetic relationships of selected taxa of the unicellular, colonial and multicellular vovlocaleans. (TIF) [file pone.0057177.s001.tif]

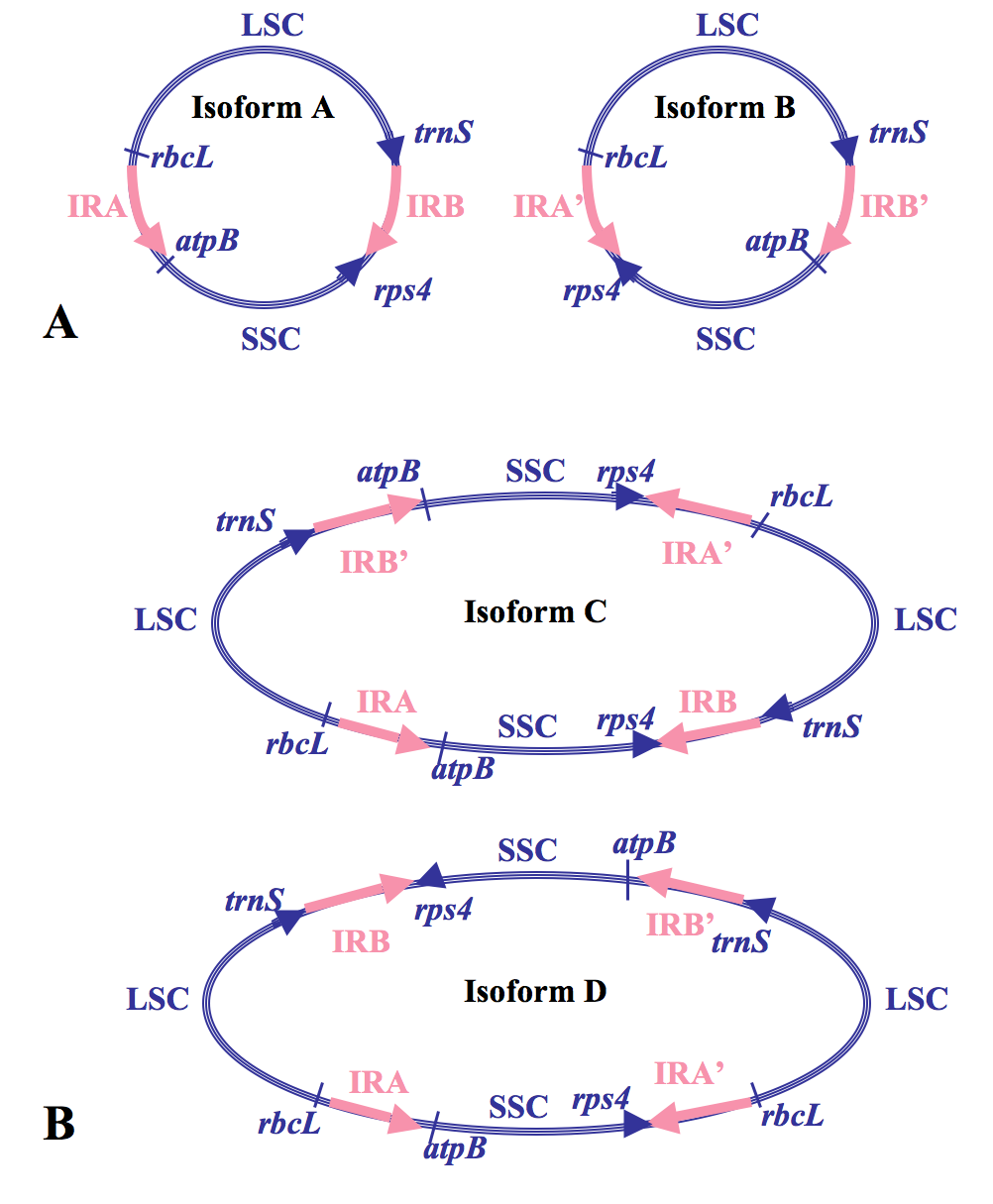

Supplement: Figure S2 — Diagrams of possible isoforms of ptDNA of Gonium pectorale. A. Two isoforms as found in other ptDNAs with a typical inverted repeat. B. Two additional isoforms that were not rejected based on assembling of our sequence data. (TIF) [file pone.0057177.s002.tif]

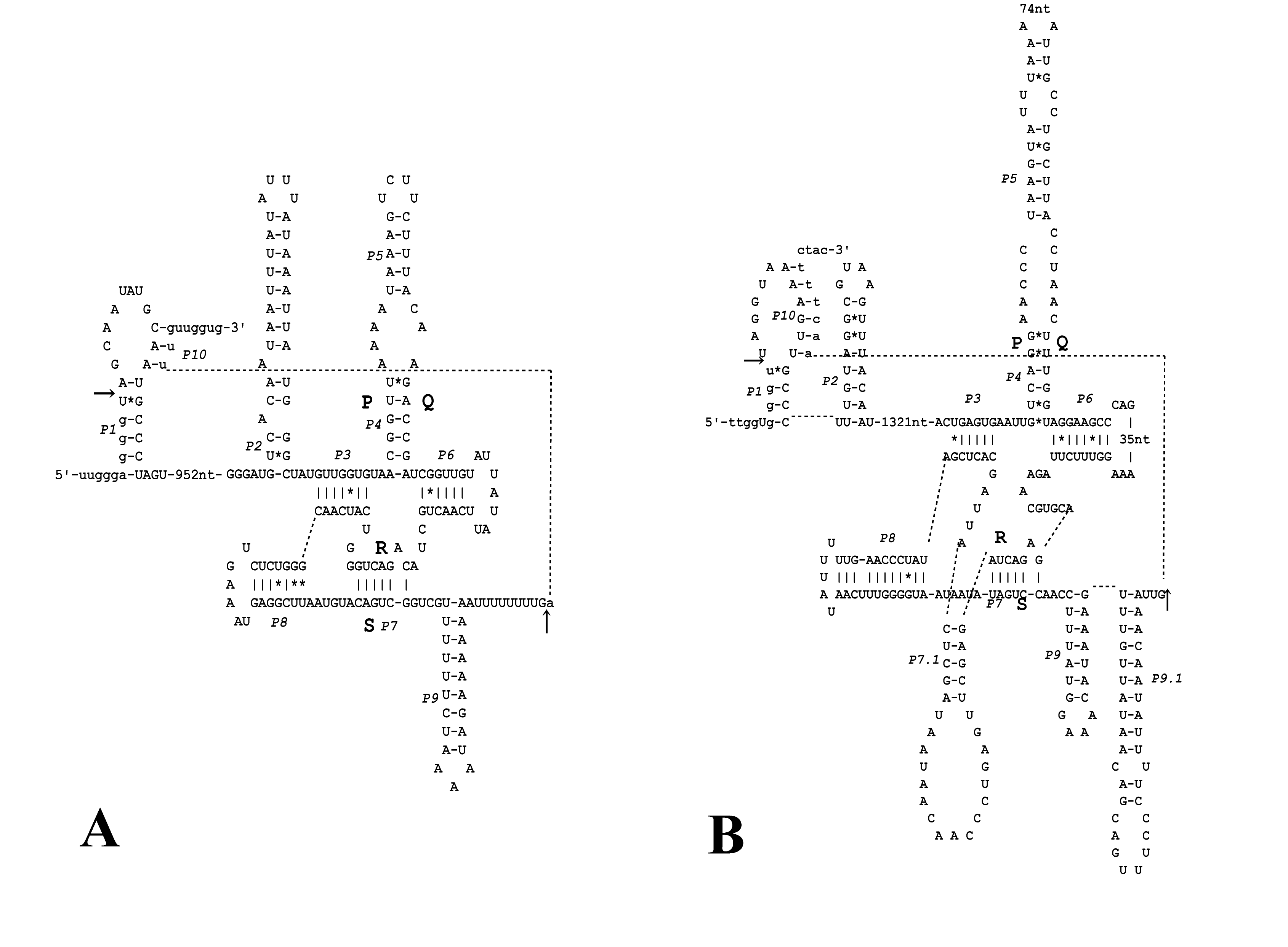

Supplement: Figure S3 — Secondary structures of group I introns within the Gonium pectorale organelle DNAs. A. Mitochondrial nad5 group ID intron. B. Chloroplast psaB group IA intron. (TIF) [file pone.0057177.s003.tif]

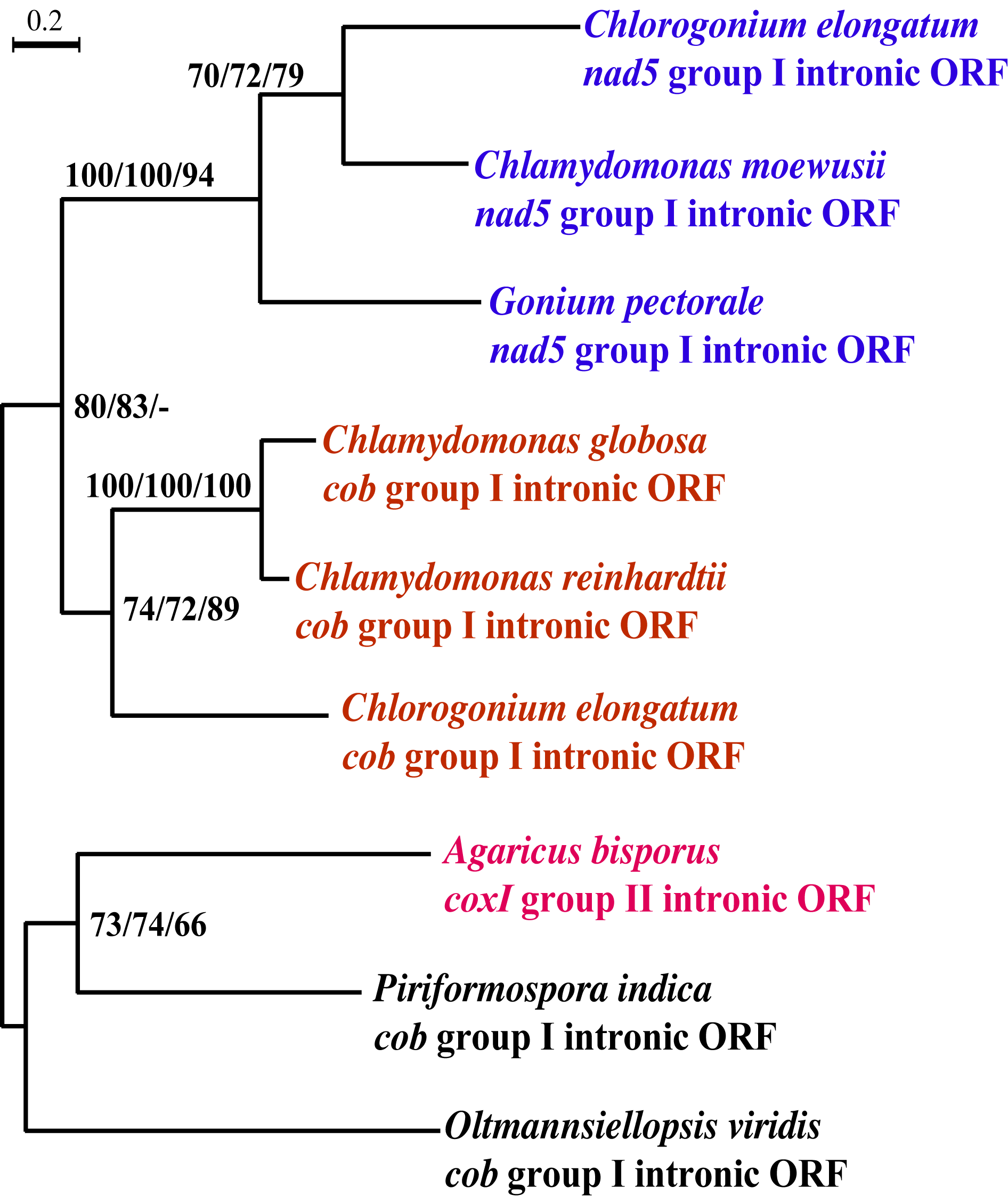

Supplement: Figure S4 — Phylogeny of Gonium pactorale nad5 group I intronic ORF. The tree was constructed under the RAxML (with WAG+4G model) method using 8 additional, related amino acid sequences selected based on the topology of the distance tree provided by blastp research of NCBI (http://www.ncbi.nlm.nih.gov/). Numbers on the left, middle and right at branches represent bootstrap values (≥50%) obtained using the RAxML, PhyML (with LG+4G model), and MP analysis, respectively. The amino acid sequences were aligned by Clustal X, and ambiguously aligned and highly variable regions were removed to construct a data matrix of 205 amino acids from the 9 operational taxonomic units (Table S2). (TIF) [file pone.0057177.s004.tif]

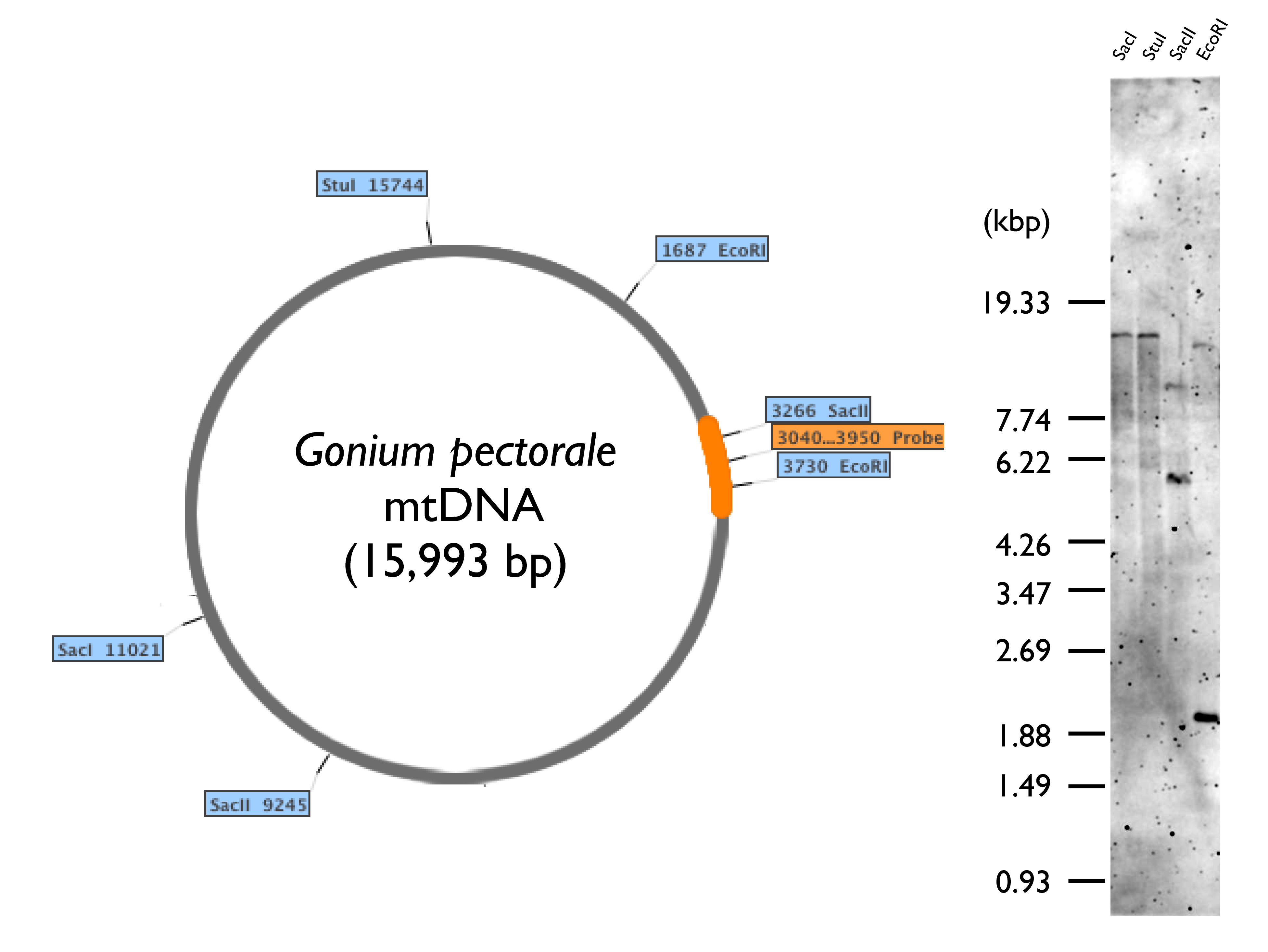

Supplement: Figure S5 — Southern blot analysis of Gonium pectorale mtDNA with four restriction enzymes that cut the genome once (SacI and StuI) or twice (SacII and EcoRI). Genome map coordinates are based on the G. pectorale mtDNA DDBJ accession (AP012493). SacI and StuI digestions each gave single genome-sized bands (∼16 kb), and the SacII and EcoRI reactions each gave two bands. These data are consistent with the G. pectorale mtDNA being a circular molecules. Probe DNA was amplified by PCR with two specific primers (Gopec-mito-F 5′-CGGGCAAAGCATAATTAGTGTAG-3′ and Gopec-mito-R 5′-ACGAACAAGAGGAAGACCTAAC-3′). (TIF) [file pone.0057177.s005.tif]

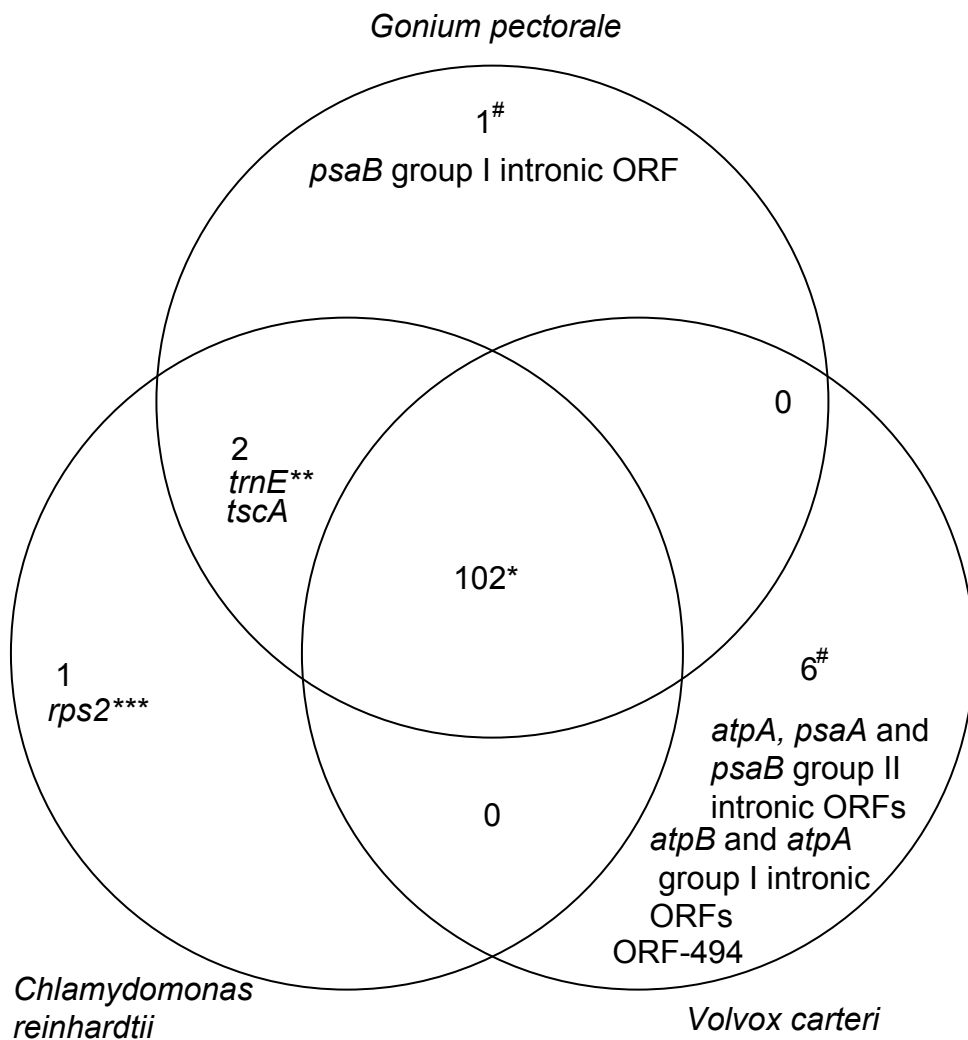

Plastid genes

Supplement: Figure S6 — Venn diagram comparing the gene repertoires of three volvocalean chloroplast genomes (AP012494, GU084820 and FJ423446). 102 genes (single asterisk) shared by the three genomes include 12 genes distributed in IRA and IRB and trnI (cau), which was previously annotated as one of the triplicated trnM in C. reinhardtii and V. carteri. Double asterisks represent one of the duplicated genes in G. pectorale and C. reinhardtii. Triple asterisks exhibit one of the duplicated genes in C. reinhardtii. Note that all intronic ORFs in G. pectprale (1#) and V. carteri (6#) are unique for each genome and considered “non-coding” in the text. (PDF) [file pone.0057177.s006.pdf]

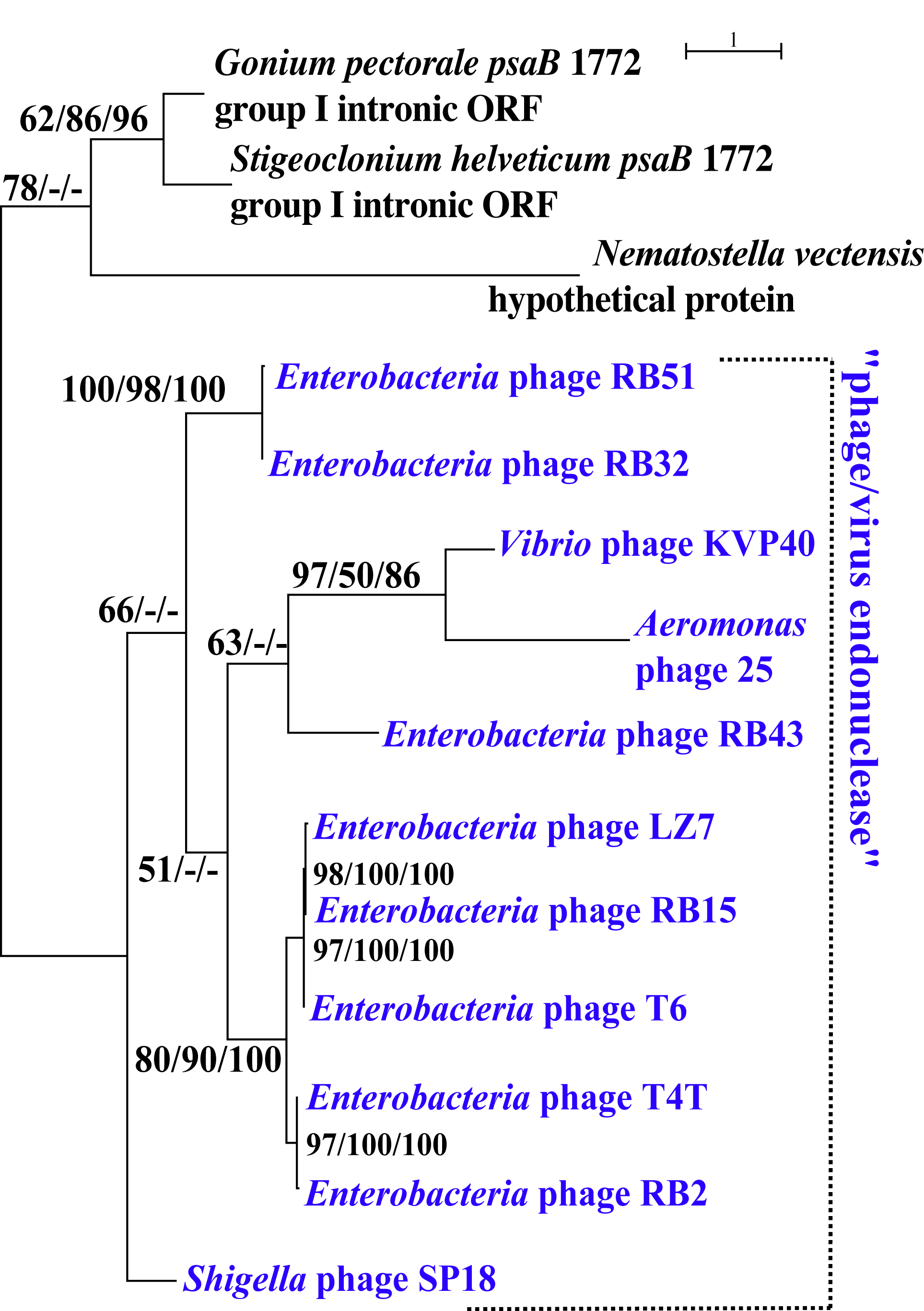

Supplement: Figure S7 — Phylogeny of Gonium pactorale psaB group I intronic ORF. The tree was constructed under the RAxML (with WAG+4G model) method using 13 additional, related amino acid sequences selected based on the topology of the distance tree provided by blastp research of NCBI (http://www.ncbi.nlm.nih.gov/). Numbers on the left, middle and right at branches represent bootstrap values (≥50%) obtained using the RAxML, PhyML (with LG+G model), and MP analysis, respectively. The amino acid sequences were aligned by Clustal X, and ambiguously aligned and highly variable regions were removed to construct a data matrix of 256 amino acids from the 14 operational taxonomic units (Table S3). (TIF) [file pone.0057177.s007.tif]

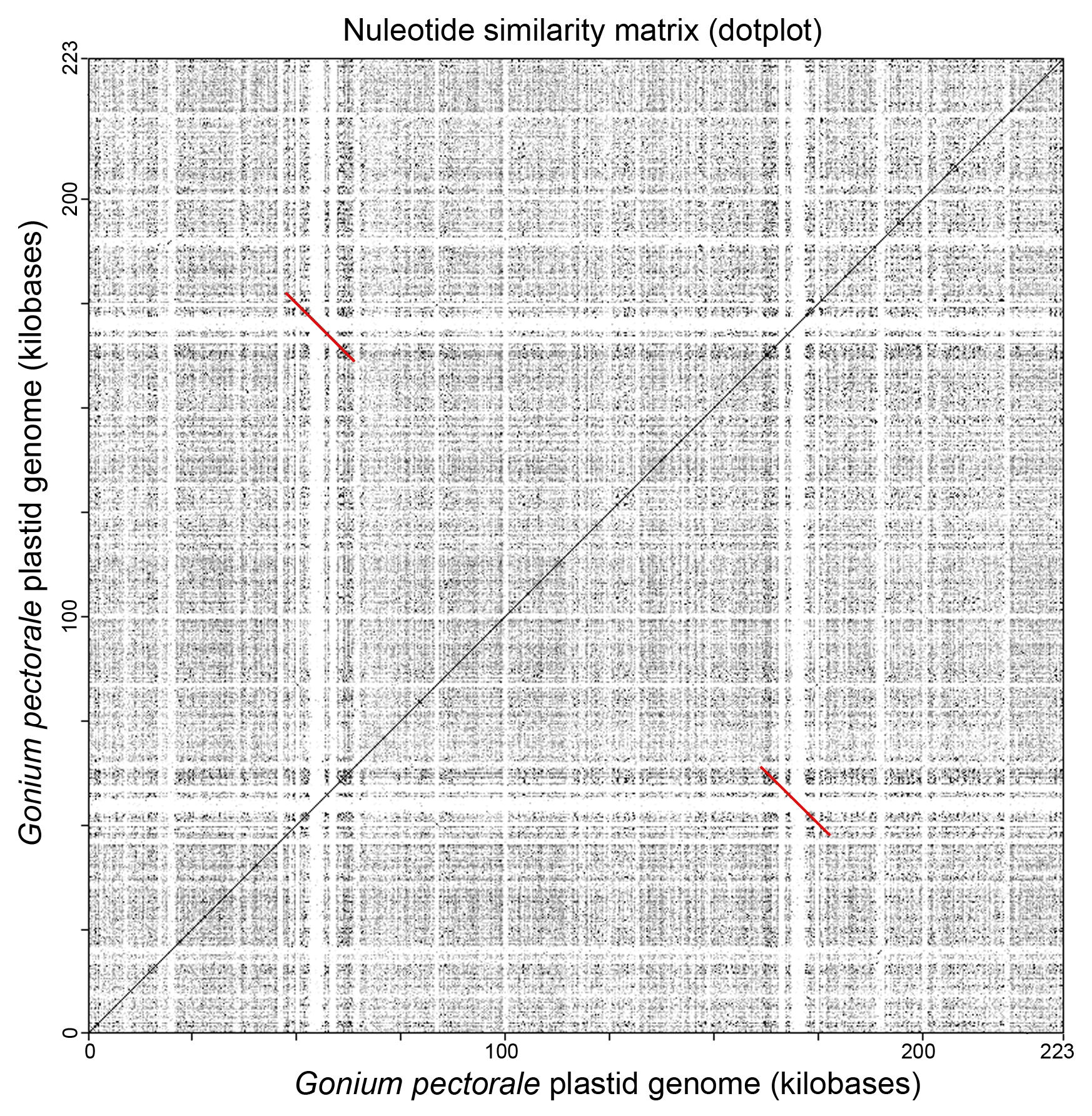

Supplement: Figure S8 — Dotplot similarity matrix of the Gonium pectorale plastid genome. The X- and Y-axes each represent the G. pectorale plastid genome (222.6 kb). Dots in the nucleotide similarity matrix represent regions of sequence similarity. The matrix was generated using JDotter, with a sliding-window size of 50. The inverted repeats are highlighted in red in the matrix. (TIF) [file pone.0057177.s008.tif]

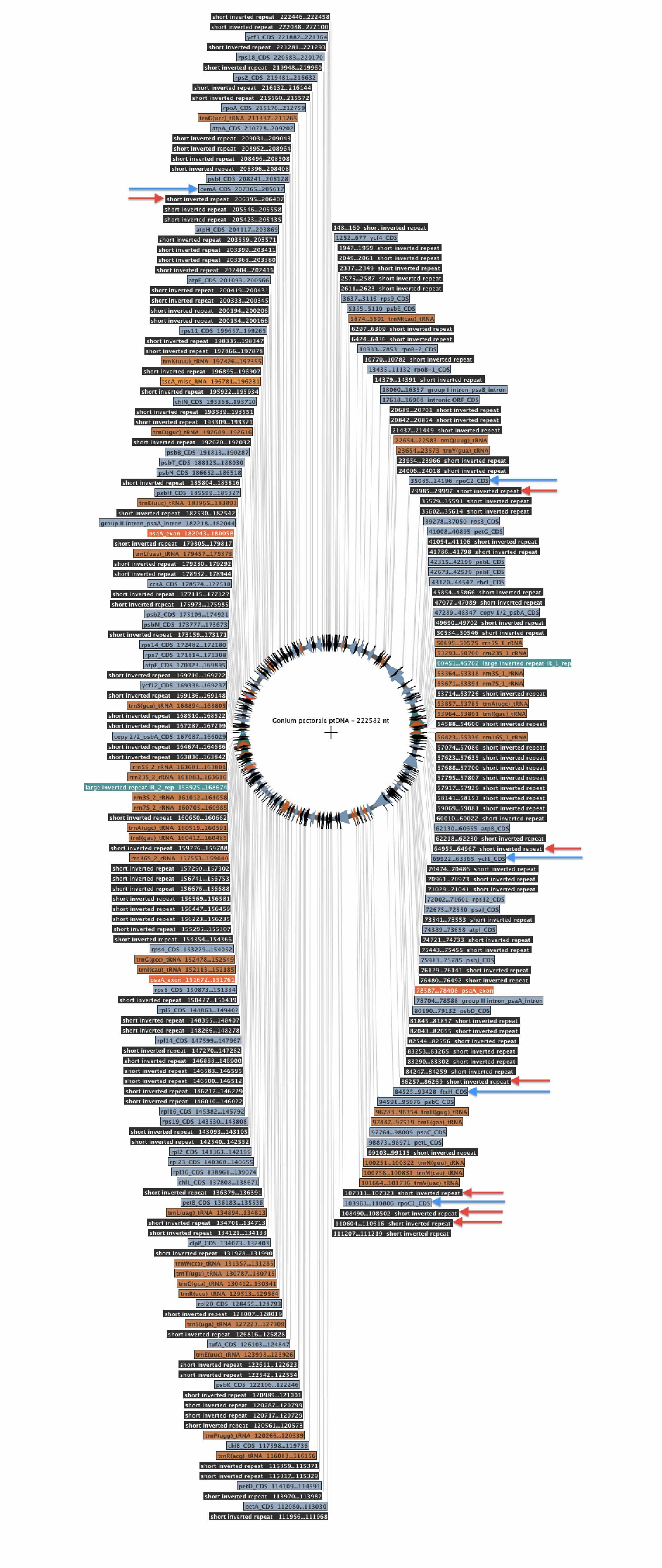

Supplement: Figure S9 — Distribution of short (13 nt) palindromic repeats (including seven [red arrows] in five coding regions [blues arrows]) with the motif: 5′- TCCCCNNNGGGGA-3′ in ptDNA of Gonium pectorale. The repeats were examined by using Serial Cloner 2.5 (http://serialbasics.free.fr/Serial_Cloner.html). (JPG) [file pone.0057177.s009.jpg]
