# Supplementary material for: Mitochondrial and Plastid Genomes of the Colonial Green Alga Gonium pectorale Give Insights into the Origins of Organelle DNA Architecture within the Volvocales
Source: PLoS One. 2013 Feb 26;8(2):e57177. doi: 10.1371/journal.pone.0057177 (PMC3582580; doi:10.1371/journal.pone.0057177)
Supplement: Table S1 — Amino acid alignment and origin of the data used for Figure 2. (DOC) [file pone.0057177.s010.doc]

Table S1. Amino acid alignment and origin of the data used for Figure 2.

Phylip alignment:

10 909

CgxElong TVTSVSTAVHCYAVVYMRSDPHLNLFLSYLSLFTFFMLVLVCSESLINMLVGWEGIGVCS

DunxSali TVSVVSFAVHCYSMVYMKADPHLNLFISYLSLFTFFMVVLISANNMLGMFIGFEGIGICS

VxCN TVTSVGFAVHMYACDYMRQDPHLSLFLGYLSLFTGFMCVLVAADNLLVMLVGYEGIGVCS

PolSAG TVVWISCAVHAYQNLYMRGDGSQTLFISYLSAFTGFMLILVAGQNLIMLFIGWEGIGVCS

PolxParv TVVWISCAVHAYQNLYMRGDGSQTLFTSYLSAFTGFMLILVAGQNLVMLFIGWEGIGVCS

PolxC TVVWVSFAVHLYQSAYMAGDSSQNLFVAYLSAFTGFMLVLVAADNLVVLFIGWEGIGVCS

Chdxince TVTGVSMAVHMYACDYMRQDPHLNLFLGYLSYFTGFMCVLVAADNLLVMLVGWEGIGVCS

GP TVTGVSLAVHMYACDYMRQDPHLSLFLGYLSFFTGFMCVLVAADNLLVMLVGWEGIGVCS

CdxRein TVTGVSMAVHMYACDYMRQDPHLNLFLGYLSYFTGFMCVLVAADNLLVMLVGWEGIGVCS

CdxMoew TVTSVSLAVHMYAVVYMRNDPHLSLFMSYLSLFTFFMLVYVSGSNLLVMLVGWEGIGVCS

YLLIGYYSHRLSATKSAQKAILVNRVSDGGAMGKSAQILFHVWLADAMEGPTPVSALIHA

YLLIGYWSHRLAASKSALKAIVVNRISDGGCMAKSVQIIFHVWLADSMEGPTPVSALIHA

YLLIGYWSHRLSAVKSAQKAILVNRISDGGAMGKSAQILFHVWLPDSMEGPTPVSALIHA

YLLIGYYGGRVSATKSANKSLIVNKISDGGAIGKSSQLFFHVWLADAMEGPTPVSALIHA

YLLIGYYGSRVSAVKSANKSLIVNKISDGGAIGKSSQLFFHVWLADAMEGPTPVSALIHA

YLLIGYYASRLAATKSANKSLIVNKVSDGGAIGKSSQLGFHVWLADAMEGPTPVSALIHA

YLLIGYWSHRLSAVKSAQKAILVNRVSDGGAMGKSAQILFHVWLADAMEGPTPVSALIHA

YLLIGYWSHRLSAVKSAQKAILVNRISDGGAMGKSVQILFHVWLPDSMEGPTPVSALIHA

YLLIGYWSHRLSAVKSAQKAILVNRVSDGGAMGKSAQILFHVWLADAMEGPTPVSALIHA

YLLIGYYSHRLAAVKSAQKAILVNRVSDGGAMGKSAQILFHVWLADAMEGPTPVSALIHA

ATLVTSGVYVMAGSLTALMAGIFGFFQSDLKRVIAFSTCSQLGYMMVSIGLGEHGADAAM

ATLVTAGIYVMVGSLTAIMGGVFGIFQNDLKRVIAFSTCSQLGYMMVSVGLGEFGVEASM

ATLVTAGVYLLVGCLTAFMAALFGATQNDLKRVIAFSTCSQLGYMMVSLGLGEIGAEASM

ATLVTAGIYVLCGATTALMGGLFGLAANDLKRVIAFSTCSQLGYMMAVLSICDDGADFAM

ATLVTAGIYVLCGAATALMGGLFGLAANDLKRVIAFSTCSQLGYMMAVLSTCDDGADFAM

ATLVTAGVYLLCGAITALAGGLFGMVANDLKRVIAFSTCSQLGYMIAAIGSCSDGMDFAM

ATLVTAGVYLLVGSLTAFMAGVFGATQSDLKRVIAYSTCSQLGYMMVSLGLGETGGEASM

ATLVTAGVYLLVGSLTAFMAAVFGATQSDLKRVIAFSTCSQLGYMFVSLGLGETGAEASM

ATLVTAGVYLLVGSLTAFMAGVFGATQSDLKRVSAYSTCSQLGYMMVSLGLGETGGEASM

ATLVTAGVYLMVGSLTALMAGIFGFFQADLKRVIAFSTCSQLGYMMVSVGLGSYGAEASM

CHLMTHASFKAALFLAAGVIIGLPETSGFYSKETIINLTRWLYSTSHKDIGLLYLGFALF

SHLMTHASFKAGLFLAAGVVIGLPETSGFYSKETIINLYRWLFSVSHKDIGILYLSFALF

GHLMSHASFKAVLFLAAGMVIGFPELSGFYSKETILNLMRWLYTTSHKDIGLLYLIFAFV

GHLVSHAGFKATLFLSAGLSIGFPELGGFYSKESILNNMRWLYSTNHKDIGMLYIIFAFF

GHLVSHAGFKATLFLSAGLSIGFPELGGFYSKESILNNMRWLYSTNHKDIGMLYLIFAFF

AHLMSHAGFKATLFLAAGLLIGFPELGGFYSKESILNTMRWLYSTNHKDIGILYLVFAFF

GHLMTHASFKAALFLAAGMVIGWPELSGFYSKETILNLMRWLYTTSHKDIGLLYLVFAFF

GHLMTHASFKAALFLAAGMVIGFPELSGFYSKETILNLMRWLYTTSHKDIGLLYLIFAFV

GHLMTHASFKAALFLAAGMVIGWPELSGFYSKETILNLMRWLYSTSHKDIGLLYLVFAFF

THLMTHASFKAALFLAAGVIIGFPETSGFYSKEAIMNLIRWLYSTNHKDIGILYLVLALF

SGLIGTSLSMFIRLELGVAGRGLLDGNFQLYNVIITGHGLIMLLFMVMPALFGGFGNWLV

AGLVGTSLSMFIRLELGLPGRGLLDGAGQLYNVIITAHGIIMLLFMVMPALFGGFGNWLV

GGLIGTSLSMLIRYELALPGRGLLDGNGQLYNVIISGHGIIMLLFMVMPALFGGFGNWLL

GGLVGTGLSVLIRLQLATTGTGILQNNGQLFNVIVTGHGVIMLLFMVMPALFGGFGNYLL

GGLVGTGLSVLIRLQLATTGTGILQNNGQLFNVIVTGHGVIMLLFMVMPALFGGFGNYLL

SGLIGTSLSIVIRLQLATTGTGLLANNGQLYNVIVTGHGVIMLLFMVMPALFGGFGNYLV

GGLLGTSLSMLIRYELALPGRGLLDGNGQLYNVIITGHGIIMLLFMVMPALFGGFGNWLL

GGLIGTSLSMLIRYELALPGRGLLDGNGQLYNVIITGHGIIMLLFMVMPALFGGFGNWLL

GGLLGTSLSMLIRYELALPGRGLLDGNGQLYNVIITGHGIIMLLFMVMPALFGGFGNWLL

AGIIGTTLSMFIRLELGLPGSGLLNGNGQLYNVIITGHGIIMLLFMVMPALFGGFGNWLV

PILIGAPDVAFPRLNNISFWLNPPALVLLLLSTLVEQGAGLGWTAYPPLSVQHSGASVDL

PIMIGAPDVAFPRLNNISFWLNPPAFFLLILSTLVEQGAGLGWTAYPPLSIQHSGAAVDL

PIMIGAPDMSFPRLNNISFWLNPPALALLLLSTLVEQGPGTGWTAYPPLSVQHSGASVDL

PLMIGAPDMAFPRLNNISFWLNPFGLLLLLVSTLVEQGAGTGWTLYPPLSVQGSGSSIDL

PLMIGAPDMAFPRLNNISFWLNPFGFLLLLVSTLVEQGAGTGWTLYPPLSVQGSGSSIDL

PLMIGAPDMAFPRLNNVSFWLNPFALLLLLLSTLVEQGAGTGWTMYPPLSVQGSGSSIDL

PIMIGAPDMAFPRLNNISFWLNPPALALLLLSTLVEQGPGTGWTAYPPLSVQHSGTSIDL

PIMIGAPDMAFPRLNNISFWLNPPALALLLLSTLVEQGPGTGWTAYPPLSVQHSGSSVDL

PIMIGAPDMAFPRLNNISFWLNPPALALLLLSTLVEQGPGTGWTAYPPLSVQHSGTSVDL

PILIGAPDMAFPRLNNISFWLNPSALGLLLLSTMVEQGAGTGWTAYPPLSIQSTGAAVDL

AIFALHINGLSSILGSINLLVTVAGMRAAGMKLNQLPLFVWAIVFTAVLVILAFPVLSAA

AILSLHLNGMSSILGSINLLVTVAGMRGTGMKANQIPLFVWSIVFTAILVILSVPVLAAA

AILSLHLNGLSSILGSVNMLVTVAGLRAPGMSLLHIPLFVWAISFTAVLVILSVPVLAAA

AILSLHLNGLSSILGSINVLVTAKGLRAPGMSLIQIPLFVYSMVFTAILVILSVPVLAAA

AILSLHLNGLSSILGSINVLVTAKGLRAPGMSLIQIPLFVYSMVFTAILVILSVPVLAAA

AILSLHLNGLSSILGSVNILVTVKGLRAPGMALIQIPLFVYSMVFTAILVILSVPVLAAA

AILSLHLNGLSSILGAVNMLVTVAGLRAPGMKLLHMPLFVWAIALTAVLVILAVPVLAAA

AILSLHLNGLSSILGAVNMLVTVAGLRAPGMKLLHIPLFVWAISFTAVLVILAVPVLAAA

AILSLHLNGLSSILGAVNMLVTVAGLRAPGMKLLHMPLFVWAIALTAVLVILAVPVLAAA

AILSLHLNGLSSILGSINILVTIAGMRAIGMKLSQMPLFVWSIAFTAILVILAVPVLAAA

LVMLLTDRNINTAYFSDSGDLVLYQHLFWFFGHPEVYILILPAFGIISHVVSFFSQKPVF

LVMLLTDRNLNTAYFCESGDLVLYQHLFWFFGHPEVYILILPAFGIVSHVVSFFSQKPVF

LVMLLTDRNLNTAYFVDSGDLILYQHLFWFFGHPEVYILILPAFGIVSHLVSFFSQKPVF

LIMLLTDRSLNTAYFVDSGDLLLYQHLFWFFGHPEVYILILPAFGLISSIISFFSNKPVF

LIMLLTDRSLNTAYFVDSGDLLLYQHLFWFFGHPEVYILILPAFGLISSIISFFSNKPVF

LIMLLTDRSLNTAYFVDSGDLLLYQHLFWFFGHPEVYILILPAFGLISSIISFFSNKPVF

LVMLLTDRNINTAYFCESGDLILYQHLFWFFGHPEVYILILPAFGLISQVVSFFSQKPVF

LVMLLTDRNLNTAYFCESGDLILYQHLFWFFGHPEVYILILPAFGIISHIVSFFSQKPVF

LVMLLTDRNINTAYFCESGDLILYQHLFWFFGHPEVYILILPAFGIVSQVVSFFSQKPVF

LVMLLTDRNLNTAYFCESGDLILYQHLFWFFGHPEVYILILPAFGIVSHVISFFSQKPIF

GVLGMICAMGAISILGFIVWAHHMFTVGLDLDTVAYFTCATMIIAVPTGMKIFSWLATIY

GVTGMIAAMGAITTLGFIVWAHHMFTVGLDLDTIAYFTSATMIIAVPTGMKIFSWLATIY

GLTGMICAMGAISLLGFIVWAHHMFTVGLDLDTVAYFTSASMIIAVPTGMKIFSWMATIY

GVTGMICAMGAIGLVGFLVWAHHMYVVGMDLDTVAYFTSASMIIAIPTGMKVFSWMATAY

GVTGMICAMGAIGLVGFLVWAHHMYVVGMDLDTVAYFTSASMIIAIPTGMKVFSWMATAY

GVTGMICAMGAIGLVGFLVWAHHMYVVGMDLDTVAYFTSASMIIAIPTGMKVFSWMATAY

GLTGMICAMGAISLLGFIVWAHHMFTVGLDLDTVAYFTSATMIIAVPTGMKIFSWMATIY

GLTGMICAMGAISLLGFIVWAHHMFTVGLDLDTVAYFTSATMIIAVPTGMKIFSWMATIY

GLTGMICAMGAISLLGFIVWAHHMFTVGLDLDTVAYFTSATMIIAVPTGMKIFSWMATIY

GNMGMICAMGAISILGFIVWAHHMFTVGLDLDTIAYFTSATMIIAVPTGMKIFSWLATIY

AGRTWFTTPMWFAVGFLSLFTIGGVTGVVLANAGVDMLVHDTYYVVAHFHYVLSMGAVFG

SGRTWFATPMLFALGFIALFTIGGVTGVVLANAGVDLLVHDTYYNISHFHYVLSMGAVFG

SGRVWLTVPMWFAIGFICLFTVGGVTGVVLANAGVDMLVHDTYYVVAHFHYVLSMGAVFG

AGKVYFSVPMLYAFGFLALFTIGGVTGVVLANAGVDTAVHDTYYVVAHFHYVLSTGAVFA

AGKVYFSVPMLYAFGFLALFTIGGVTGVVLANAGVDTAVHDTYYVVAHFHYVLSTGAVFA

AGKVYFSVPMLYAFGFLALFTIGGVTGVVLANAGVDTAVHDTYYVVAHFHYVLSTGAVFA

SGRVWFTTPMWFAVGFICLFTLGGVTGVVLANAGVDMLVHDTYYVVAHFHYVLSMGAVFG

SGRAWFTAPMWFAVGFICLFTLGGVTGVVLANAGVDMLVHDTYYVVAHFHYVLSMGAVFG

SGRVWFTTPMWFAVGFICLFTLGGVTGVVLANAGVDMLVHDTYYVVAHFHYVLSMGAVFG

GGSLWLTTPMWFAVGFICLFTLGGVTGVVLANAGVDMLVHDTYYVVGHFHYVLSMGASFG

IFAGLYFWFLMTGLSYYEGRGQVHFWTLFIGVNLTFFPMHMLGLAGMPRRMFDYADAFIG

IFAGLYFWLLMTGLSYNEARGHLHFYLLFIGVNLTLYKRVDIVLAGAPRRVFDHPDSFAG

IFGGLYFWGLITGLGYNEGRAMVHFWLLFIGVNLTFFPLHFLGLSGMPRRMFDYADCFAG

IFAGMYFYSLMFNLGYDENKGTVQFLLFFLGVNLTFFPQHFLGLAGMPRRMFDYADGFTG

IFAGMYFYSLMFNLGYDENKGTVQFLLFFLGVNLTFFPQHFLGLAGMPRRMFDYADAFTG

IFASIYFYSLIFHLGYDESKGTVQFLLFFLGVNLTFFPQHFLGLAGMPRRMFDYADAFSG

IFAGVYFWGLITGLGYHEGRAMVHFWLLFIGVNLTFFPQHFLGLAGMPRRMFDYADCFAG

IFGGLYFWGLITGLGYHEGRAMVHFWLLFIGVNLTFFPQHFLGLAGMPRRMFDYADCFAG

IFAGVYFWGLITGLGYHEGRAMVHFWLLFIGVNLTFFPQHFLGLAGMPRRMFDYADCFAG

IFAGIYFCQFKTGLTYIESRGQVQFWTLFIGVNLTFFPMHMMGLGGMPRRMFDYADCFYG

WNSLASYGAMVSFLSLLLLAGPVSLVPQGAPQTATTLEWLLPATPANHTFSQLPVLRTTY

WNALASYGSIISFLSVLLLAGPISLVPQHTPSASSSLEWLLPATPANHTFNQLPVLRATY

WNSVSSFGASISFISLFVLATTFQDAVRTVPRTATTLEWVLPATPANHVFSQVPVLRTTY

LNLLSSYGALVSFTSILYAGTVFTPAPALQNRTSTSLEWLLPATPAFHTFSEVPVLRVAY

LNLLSSYGALVSFTSLLYAGTVFTPAPALQNRTSTSLEWLLPATPAFHTFSEVPVLRVAY

LNALSSYGAMISLTSILYVGTVFTPAPAFQNQTATSLEWILPATPAFHTFKEVPVIRNAY

WNAVSSFGASISFISVLVFATTFQEAVRTVPRTATTLEWVLPATPAHHVFSQVPVLRTTY

WNAVSSFGASISFISVLVLATTFQEAVRTTPRTAVTLEWLLPATPANHVFSQVPVLRSSY

WNAVSSFGASISFISVIVFATTFQEAVRTVPRTATTLEWVLLATPAHHALSQVPVLRTAY

WNSIASFVALISFLSILMLAGPINFMPEIYPRSATTLEWMDHSTPASHVFMQLPVIRSYY

PTPLNLNYAFKFGSLAGVVLMVQIVTGILLAMHYTAHVDHAFSSVIHLMNDVPSGMILRY

PTPMNLSYAYNFGSLAGIVLASQIITGILLAMHYVGHVDLAFNSVVHLMNDVPSGMILRY

PTPMNLNYSWNWGSLAGLMLASQLVTGILLAMHYVGHVDYAFASVQHIMTDVPSGIILRY

PTPCNLKYSWNMGSLSGLLLASQIVTGILLAMHYCPDTSLAFYSVIHLTVDVPYGFVIRY

PTPCNLKYSWNMGSLSGLLLASQIVTGILLAMHYCPDTSLAFYSVIHLTVDVPYGFVIRY

PTPANLKYSWNMGSLSGLLLAGQIVTGVLLAMHYCPDTTLAFASVLHLTVDVPYGFVIRY

PTPMNLNYSWNGGSLAGMMLASQMLTGILLAMHYVGHVDYAFASVQHLMTDVPSGMILRY

PTPMNLNYSWNGGSLAGMMLASQMLTGILLAMHYVGHVEHAFASVQHLMTDVPSGMILRY

PTPMNLNYSWNGGSLAGMMLASQMLTGILLAMHYVGHVDYAFASVQHLMTDVPSGMILRY

PTPLNLNWSWSWGSLSGLVLASQIVTGILLAMHYVGHVDHAFASVQHLMVDVPSGVILRY

THANGASLFFTVVYLHTFRGIYYSSGNQPRELVWITGVVILLVMIITAFIGYVLPWGMSL

AHANGASLFFIVVYIHILRGVYYSSGNQPREAVWITGVVILLVMVLTAFIGYVLPWGSLG

AHANGASLFFIVVYVHILRGFYYGSGTQPRELVWITGVIILLVMIITAFIGYVLPWGMSF

FHMNGASLFFVAVFLHLFRNLYYNSGSQPRELLYISGVIILLLMVITAFIGYVLPWGMSF

FHMNGASLFFVAVFLHLFRNLYYNSGSQPRELLYISGVVILLLMVITAFIGYVLPWGMSF

FHMNGASLFFVAVYLHLFRNLYYNSGSQPREVLYISGIVILLLMVITAFIGYVLPWGMSF

AHANGASLFFIVVYLHVLRGMYYGSGAQPREIVWISGVIILLVMIITAFIGYVLPWGMSF

AHANGASLFFIVVYLHILRGMYYGSGAQPREMVWISGVVILLLMIITAFIGYVLPWGMSF

AHANGASLFFIVVYLHVLRGMYYGSGAQPREIVWISGVVILLVMIITAFIGYVLPWGMSF

THANGASLFFTVVYLHVLRGLYYSSGNQPREIVWISGVVILLLMVITAFIGYVLPWGMSF

WGATVITSLATVIPVVGKDIVTWLWGGFSIDNPTLNRFYSFHYTLPFILAGLSIFHIAAL

PIATVITSLATVIPVVGKTVLSYLWGGFSVDNPTLNRFYSLHYTFPFVLAGLSIFHIAAL

WGATVITSLATAIPVVGKHIMYWLWGGFSVDNPTLNRFYSFHYTLPFVLAGLSVFHIAAL

WGATVITSLVSAVPIVGTDLVYYLWGGFSVSNPTLNRFFSFHYLLPFVLAGLSIAHLAAL

WGATVITSLVSAVPIVGTDLVYYLWGGFSVSNPTLNRFFSFHYLLPFVLAGLSIAHLAAL

WGATVITSLVSAVPVMGTALVYYLWGGFSVSNPTLNRFFSFHYLLPFVLAGLSLAHLAAL

WGATVITSLATAIPVVGKHIMYWLWGGFSVDNPTLNRFYSFHYTLPFILAGLSVFHIAAL

WGATVITNLASAIPVVGDAIKYWLWGGFSIDQPTLNRFYSLHYTLPFVLAGLSIFHIAAL

WGATVITSLATAIPVVGKHIMYWLWGGFSVDNPTLNRFYSFHYTLPFILAGLSVFHIAAL

WGATVITSLVTTIPIVGKQIVFWLWGGFSIDHPTLNRFYSLHYTLPFVLAGLSIFHIAAL

HQYGSTNPIPANPYVTPQHIVPEWYFLWVYAILRSIPNK

HQYGSTNPIPANPYSTPQHIVPEWYFLWFYAILRSIPNK

HQYGSTNPIPANPYSTPQHIVPEWYFLWVYAILRSIPNK

HSYGSTNPIPANPYSTPAHIVPEWYFLPVYAILRSIPDK

HSYGSTNPIPANPYSTPAHIVPEWYFLPVYAILRSIPDK

HSYGSTNPIMANPYSTPAHIVPEWYFLPVYAILRSIPDK

HQYGSTNP-------------------------------

HQYGSTNPIPANPLVTPHHIVPEWYFLWVYAILRSIPSK

HQYGSTNPIPANPYSTPQHIVPEWYFLWVYAILRSIPNK

HQYGSTNPIPANPYSTPQHIVPEWYFLWVYAILRSIPNK

Taxa and accession numbers:

*Chlorogonium elongatum* (CgxElong) Y13643

*Dunaliella salina* (DunxSali) NC_012930

*Volvox carteri* (VxCN) EU760701

*Polytomella* sp. SAG 63-10 (PolSAG) NC_013472

Polytomella parva (PolxParv) NC_016916

*Polytomella capuana* (PolxC) NC_010357

*Chlamydomonas globosa* (Chdxince) DQ373068

*Gonium pactorale* (GP) AP012493

*Chlamydomonas reinhardtii* (ChCdxRein) CAA38641 (*nad5*), ABX82077 (cob), NP_042567 (coxI)

*Chlamydomonas moewusii* (CdxMoew) NC_001872
