# Supplementary material for: Mitochondrial and Plastid Genomes of the Colonial Green Alga Gonium pectorale Give Insights into the Origins of Organelle DNA Architecture within the Volvocales
Source: PLoS One. 2013 Feb 26;8(2):e57177. doi: 10.1371/journal.pone.0057177 (PMC3582580; doi:10.1371/journal.pone.0057177)
Supplement: Table S2 — Amino acid alignment and origin of the data used for Figure S4. (DOC) [file pone.0057177.s011.doc]

Table S2. Amino acid alignment and origin of the data used for Figure S2.

Phylip alignment:

9 205

GPnad5 IPANKRHG-LHCFLFKQLLVGFMLGDGWLEKHGKGVRLAISLTEKFKDVAQFYLVLLYGL

CdGlcob VPADKRIG-PHTAEFCDIAVGLLLSDAHLEQHGKGVRLTFSQSIVHVQFFEQTAKNLQRL

CgElcob IKAKNRHG-PHRWEFVQVAVGLLLSDAHAEVHGNGVRISFQQEKTFADYFSFVYGILERL

CdMnad5 GATLASSGFPHSFLFKQILTGLLLGDGWLERHGKGTRLGVSCKHVYADVANWMQLMFYGL

CgEnad5 --------------MHQIVVGCLLGDGHLERRGN-TRLGISFKHVYKDVADWYLTMFYGL

CdRcob VPALKRIG-PHTAEFCDIAVGLLLSDAHAERHGKGVRLTFSQSTSHRPFFEHTAKALQRL

Oltman KLDIQKLLESLDQETVSVLTGLILGDASCESRSLGTARFFKQESSNVEYLMKIHKFLAKK

Agaric QKLEANIKPSISIEVKEILFGSLLGDGKLELPPRGLNARFIQSLEKKDYFLSVLNSLGDL

Pirifo -------------MWGTKIPGIILNS---------VRFALEQSIKNNAYLISVWTELKLL

GYTNKFELGLPLVRKNKTTKP--YYQIKTFTFESLLPYFSLWYKQMIKQLPEQLYELLSP

GYVNEKAYVP-LQTTDSNGKVHYYLKCHTFVCASLSWLQDLFYVGNTKVVKPELEPFLTP

GYVTRKGVSDGVSFHTRNQGARTYFRLNTFTFSSLIWLRELFYDANVKVIRPELINYLTP

GYHDKMYQVSPLECITRQGKISRYYQVRTFSFASLNKYYNLWYVNNIKIVPLDIDQYLTP

GYQDNIAFENYSTRTYPSGRVYKYYQVRTRSYSNFNILYERWYPSRVKVVPAYIVEFLTP

GYVHEKTYTP-LPTTDSNGKVCEYLRLHTFVFGSLSWLRDLFYVDNIKVVKPELEPFLTP

KIVNPSKPKLR-TRVVKNSKVRFYYRLNTFTRKSLFVFYKGFYIRIVKIIPENIIEVLDL

CSGKYREYSY---FDKRTGKTYTSLNFWTRALPVLNEFNLKFYSPVIKIVP-QDLSLLTP

GYCSDK-----SPITITKPNGLSNIRFYLFTFTSLFWIFEGFYTFVIKSVPVWIELYFSP

FTLAVWIMGDGSGMRDGGFKIASHSFTKEQNMVLCNILAERYNIKTTLVNEKGLYHIYVW

RALAYWIAGDGA-WDNHGLRLSTHNFKYDECIMLTQMLNRRFSLKCTVQAGTVCYRIYVV

ISLRHAICGDGS-STDYGTSLSFNSFTYEECVLFTNMLKEKFGIIASVQSAPNQYRVYIQ

LALAIWLMGDGSGMRDGGFKISTHSFTKQENEFLVELLLNKYDIKASIHRDGDEFNIYIW

LALALWCMGDGSAMDCGGFKLSTHGFTYEDNLFLARTLKELYGLEVTLHRDPAA------

RALAYWIAGDGC-WDNAGLRLSTHNFKHDECVMLTHMLSRRFGLECTVQKETIYYRIYVV

KCLATWFMGDGSVGGSHGLKLATNSFYKTDVIRLSEALNTRHGFNTTVQRDGKQYIIYFP

IAIAHWTMQGGSRGTSKGLYLCTDSFTYADVLRLSKYLNNKYNLKSSIHKAGGNYRIYIL

ITIAHWFMQDGSRQAGQGVYFATNSFTYEDTTRLANLLTSKYGLKTSVIKYENQWRISIW

KRSTHLLYVIVKPYLLPSCEYKFRF

SKSMPHLRTLVTHYLDPSMHYKINN

AASMNTLRAIVLPHMPKSMHYKVI-

KQSVPKVKALVLPFFHVRRVARHAN

----PWAEAANIIIFM---------

AKSMPRLRSLVSCYLDPSMHYKINN

SRDVPEVQAKIKDLMVSSMHYKLRI

AKSVETVKILILPFMHKTMKYKLGV

KESMPRFAALIIPYMHPSMLYKLEG

Taxa and accession numbers:

*Gonium pectorale* (GPnad5) AP012493

*Chlamydomonas globosa* (CdGlcob) ABC98219

*Chlorogonium elongatum* (CgElcob) CAA69146

*Chlamydomonas moewusii* (CdMnad5) NP_046148

*Chlorogonium elongatum* (CgEnad5) CAA73987

*Chlamydomonas reinhardtii* (CdRcob) CAA39012

*Oltmannsiellopsis viridis* (Oltman) YP_684382

*Agaricus bisporus* (Agaric) ABY85445

*Piriformospora indica* (Pirifo) CCA78107
