# Supplementary material for: Mitochondrial and Plastid Genomes of the Colonial Green Alga Gonium pectorale Give Insights into the Origins of Organelle DNA Architecture within the Volvocales
Source: PLoS One. 2013 Feb 26;8(2):e57177. doi: 10.1371/journal.pone.0057177 (PMC3582580; doi:10.1371/journal.pone.0057177)
Supplement: Table S3 — Amino acid alignment and origin of the data used for Figure S7. (DOC) [file pone.0057177.s012.doc]

Table S3. Amino acid alignment and origin of the data used for Figure S4.

Phylip alignment:

14 256

Stigeo FLMDILSEKRISKTTLIYKKVIESKNSKTKAFYTKIFYVYRLTFT--HPIS---LEKFFY

RB43 MIYTKLKEWLPHD---LYVEYVDIITASPKTEYTEKHHILPRSLFPEFENDPDNLVELDV

LZ7 MNYQNVYNSLISR----------AQTREPLLGYKETHHIIPRCMG--GSDDKENLVELTG

T6 MNYQKIYNDLISR----------AQAREPLSEYKETHHIIPRCMG--GSDDKENLVELTG

T4T MNYQKIYNDLISR----------AQAREPLSEYKETHHIIPRCMG--GSDDKENLVELTA

OxRB51 MNYKLIYEKLISN----------AKSRK-LDCYTESHHIIPRCLN--GSDDASNLVDLTP

OxSP18 MNYALIYENLMNR----------AKSRK-LTGYKESHHVVPQCLG--GPDTKENRVDLTP

OxRB32 MNYKLIYEKLISN----------AKSRK-LDCYTESHHIIPRCLN--GSDDASNLVDLTP

KVP40 MAYLYKITNPVNGKEYIG---VTIDLKRRFSEHARKDYAIGQAIR-KYGITFEDMEVIAS

Aeromona MWYLYKVTNKLNGRYYVG---VHKSDNIETDPYMGSGRAIRHAITKYGISNFDREILAEF

Nematost ------------------------MEENSVESLSELFRCFICMERLQDARLCPHCSKLCC

GPpsaBOR ----MASNK---------------KTQNSQILNKKIFYTYRLTCI--DDTYKNNNTEIYY

RB2 MNYQKIYNDLISR----------AQAREPLSEYKETHHIVPKCMG--GSDDKENLVELTA

RB15 MNYQNVYNSLISR----------AQTREPLLGYKETHHIIPRCMG--GSDDKENLVELTG

YG-YRGCMAAPLADSSYWSSSKTVKQTLKEFGATCFKKKILGVYTWIDTALNREIFLHKK

MKHLMAHRTLAKTND--HKMILAFFMMF------TYEHKRYSTLSEQE------------

REHFIAHWLLCKIYD-TPGLKKAFGLMC-LTG-KNRSYKISSQLYELG------------

REHFIAHWLLCKIYD-TPGLKKAFGLMC-LTG-KNRSYKISSQLYELG------------

REHFIAHAILSKIYP-VKSVIFAFFMMCNMKGTKKRHYKVHSKIYAHAKKLNSQFRKGTV

EEHYVAHQLLVKIYPSEPKLIYAANMMTVSTKYAKRNNKTFGWIRRKLWEIEKNKEFSQE

EEHYLAHQLLVKINPEHKGLKYAAYMMTISPDGRRQNNKLFGWLKRDYLNNRIQSSGMKG

EEHYVAHQLLVKIYPSEPKLVYAANMMTVSTKYAKRNNKTFGWIRRKLWELEKNKEFSQE

GTEEEMYELESKIVTEEYVLSESNYNLVPGGIGRINGYKHTDESKRKMSEAKKG------

DCEELAYFVESEIVDAYFVDMPETYNMAIGGKGGWSHIDTKGWMRYCSANDKKKHSKSVS

FLCIRRWLTEQRPQCPHCRASVHLHELVHCRWVEEVTQQIDS------------------

MG-YRSTKTLPVLDD-YYSSSKTVKNLIASVSKTKFKKKILGLYANQTEAIENEVVYHKK

REHFIAHAILSKIYP-VKSVIFAFFMMCNMKGTKKRHYKVHSKIYAHAKKLNSQFRKGTV

REHFIAHWLLCKIYD-TPGLKKAFGLMC-LTG-KNRSYKISSQLYELG------------

FDVKNNPKFMNRANQTSTKFQFERTGIPQSEISNKKRSKALRGRVSPETRERMRQSHLN-

-----------------QQFILEEKTKAREAMRVVKKEQMKGKYDGENNPFYGKRH----

-----RRRLSEAAIG--REVSIETREKISRALKGRKFTKEHLAHMRKPKTEEAKKN----

-----RRRLSEAATG--REVSMETREKISRALKGRKFTKEHLARMRKPKTEEAKKN----

ISEETRLKMSKAKTG--LRLTEETKHKISAATKGRAKSEETKKKMRKPKTEEAKKN----

TRKKMSDAVANKAR---LTCPHCGKSGLSGNMNRWHFDNCPNHPNPKVRPPMSKEHRNNI

KKHKPESIQLMKDKRALQKITETTKAKISKTKTGVKFTKEALKSFNEKRSANQAWIDSNF

TRKKMSDAVANKAR---LTCPHCGKSGLSGNMNRWHFDNCPNHPNPKVRPPMSKEHRNNI

--TTPHNKGKKASLESRRKMADAKRGKKRGAMSDETKSKLSQANSGENHGMYGKTHS---

ESRKSSERCRKSSIENFKKASAARIGSKDSAETIEKRKMSLRQFYKETHKKALSDAWTPE

-----------------LQLTVPSKAREDADKDSSSYVN---------------------

LKVNCNLKFLNKACQTNTKFYYDNTGRIPTTESNLKRSARLLGRITPEGKARVASYQKNQ

ISEETRLKMSKAKTG--LRLTEETKHKISAATKGRAKSEETKKKMRKPKTEEAKKN----

-----RRRLSEAAIG--REVSMETREKISRALKGRKFTKEHLARMRKPKTEEAKKN----

RVRSEEETRGRREAMIRTNQQVAICPHCGLQVGMPGGRRWHFNNCLQNPNVSEDAIRDHE

--------TDEFKKMMGSVHKGKKLSPEHLANLVAAHKGKKREKVK---CPHCGIMCAAN

---IAAAKVGVLNPMYGTISPTRDVPHTKETRDLISLRTKQGTEYP--PCPHCGKKVNKG

---IAAAKVGVLNPMYGTISPTRDVPHTKETRDLISLRTKQGTEYP--PCPHCGKKVNKG

---IAAAKVGVLNPMYGTISPTRDVPHTKETRDLISLRTKQGAEYP--PCPHCGKKVNKG

SNGLKAIILEETSCEFCGMIGKKCVIAAHRNFCKKNPNGRKKTEKTG-TCVHCGKVTTFG

RPCKESTKSKIGDANRGRVFDKKTCPHCGKTGAGPN-MKRYHFDNCK-SKVAKCNLPAFN

SNGLKAIVLEETSCEFCGMIGKKCVIAAHRNFCKKNPNGRKKTEKTG-TCVHCGKVTTFG

--------EEARRKISEASRKRAENMSDEEREQKRERMKKNNPNNIKVTCPHCGKETTLP

MRLKQAERGRSSTSFIMSRLGVTLSRETKELLSASAKARWNKLPRVMIQCPHCLKEGVSH

----------IIHSVHSQKKKKKKIDRGGHFVLE--------------------------

RERTVEELNQLSKAATERNNQTATCPHCGRVGQYLAMLRWHFDRCPKAPNPSAEGIADRE

---IAAAKVGVLNPMYGTISPTRDVPHTKETRDLISLRTKQGAEYP--PCPHCGKKVNKG

---IAAAKVGVLNPMYGTISPTRDVPHTKETRDLISLRTKQGTEYP--PCPHCGKKVNKG

RVRQAQIERNKNRSRN

TAKRWHFDNCKSGLVQ

NALRWHYDKCKFKDSK

NALRWHYDKCKFKDSK

NALRWHYDKCKFKDSK

NLSRWHNDNCKHKH--

TNTTPHLDNVLPIVNG

NLSRWHNDNCKYKPS-

ASKRWHFDNCKHKS--

AMKRWHFDKCKQKKD-

----------------

KVRQNAIKRNK-KPKN

NALRWHYDKCKFKDSK

NALRWHYDKCKFKDSK

Taxa and accession numbers:

*Stigeoclonium helveticum* (Stigeo) YP_764417

*Enterobacteria* phage RB43 (RB43) YP_239192

*Enterobacteria* phage LZ7 (LZ7) ABA03236

*Enterobacteria* phage T6 (T6) ABI48942

*Enterobacteria* phage T4T (T4T) ADJ39953

*Enterobacteria* phage RB51 (OxRB51) YP_002854012

*Shigella* phage SP18 (OxSP18) YP_003934858

*Enterobacteria* phage RB32 (OxRB32)

*Vibrio* phage KVP40 (KVP40) NP_899393

*Aeromonas* phage 25 (Aeromona) YP_656271

*Nematostella vectensis* (Nematost) XP_001636201

*Gonium pactorale* (GPpsaBOR) AP012494

*Enterobacteria* phage RB2 (RB2) ABA03239

*Enterobacteria* phage RB15 (RB15) ABA03242
